# Potent and sustained huntingtin lowering via AAV5 encoding miRNA preserves striatal volume and cognitive function in a humanized mouse model of Huntington disease

**DOI:** 10.1093/nar/gkz976

**Published:** 2019-11-20

**Authors:** Nicholas S Caron, Amber L Southwell, Cynthia C Brouwers, Louisa Dal Cengio, Yuanyun Xie, Hailey Findlay Black, Lisa M Anderson, Seunghyun Ko, Xiang Zhu, Sander J van Deventer, Melvin M Evers, Pavlina Konstantinova, Michael R Hayden

**Affiliations:** 1 Centre for Molecular Medicine and Therapeutics, Vancouver, British Columbia, Canada; 2 Department of Medical Genetics, University of British Columbia, Vancouver, British Columbia, Canada; 3 BC Children's Hospital Research Institute, Vancouver, British Columbia, Canada; 4 Burnett School of Biomedical Sciences, College of Medicine, University of Central Florida, Orlando, Florida, USA; 5 Department of Research & Development, uniQure biopharma B.V., Amsterdam, the Netherlands

## Abstract

Huntington disease (HD) is a fatal neurodegenerative disease caused by a pathogenic expansion of a CAG repeat in the huntingtin (*HTT*) gene. There are no disease-modifying therapies for HD. Artificial microRNAs targeting *HTT* transcripts for degradation have shown preclinical promise and will soon enter human clinical trials. Here, we examine the tolerability and efficacy of non-selective HTT lowering with an AAV5 encoded miRNA targeting human *HTT* (AAV5-miHTT) in the humanized Hu128/21 mouse model of HD. We show that intrastriatal administration of AAV5-miHTT results in potent and sustained HTT suppression for at least 7 months post-injection. Importantly, non-selective suppression of huntingtin was generally tolerated, however high dose AAV5-miHTT did induce astrogliosis. We observed an improvement of select behavioural and modest neuropathological HD-like phenotypes in Hu128/21 mice, suggesting a potential therapeutic benefit of miRNA-mediated non-selective HTT lowering. Finally, we also observed that potent reduction of wild type HTT (wtHTT) in Hu21 control mice was tolerated up to 7 months post-injection but may induce impairment of motor coordination and striatal atrophy. Taken together, our data suggests that in the context of HD, the therapeutic benefits of mHTT reduction may outweigh the potentially detrimental effects of wtHTT loss following non-selective HTT lowering.

## INTRODUCTION

Huntington disease (HD) is a fatal neurodegenerative disease that affects ∼13.7 per 100 000 individuals in the general population (1). HD is caused by a dominant CAG trinucleotide repeat expansion mutation in the huntingtin (*HTT)* gene beyond 35, which codes for an elongated polyglutamine (polyQ) tract in the HTT protein (2). HTT is a highly conserved protein that functions as a cellular scaffold, mediating interactions with many biomolecules and organelles, and as such is involved in a plethora of cellular pathways, including transcriptional regulation (3–5), mitochondrial function (6,7), axonal trafficking (8–10), endocytosis (11,12) and cellular stress responses (13–15). In HD, the expansion of the polyQ tract leads to perturbation of these normal functions and also induces the gain of toxic functions causing cellular dysfunction and ultimately neuron death. Notably, multiple preclinical studies have demonstrated that suppressing mutant HTT (mHTT) in the brain can improve and even reverse molecular, neuropathological and behavioural abnormalities in various rodent models of HD (16–28). Therefore, reducing levels of mHTT represents a promising therapeutic strategy targeting the cause of the disease and has the potential to modify clinical progression of the disease.

Lowering mHTT can be achieved through a variety of analogous approaches, including repressing transcription of the *HTT* gene (zinc finger repressors), inducing degradation of *HTT* RNA transcript (RNA interference and antisense oligonucleotides) or promoting clearance of the HTT protein (proteasome and autophagy activators). The mechanisms and state-of-the-art of these approaches have been extensively reviewed elsewhere (29–31) and thus will not be discussed in detail here. Instead, we will focus on HTT lowering using RNA interference (RNAi).

RNAi represents a promising approach to suppress *HTT* expression as a therapy for HD. The introduction of artificial RNA into the cell that is complementary to the *HTT* mRNA can induce selective transcript degradation by exploiting the endogenous RNA interference machinery (32). This has been demonstrated using short interfering RNA (siRNA) (22,33), short hairpin RNA (shRNA) (17,18,20,21,26,27) and artificial microRNA (miRNA) (19,24,34–36) targeting *HTT* in various models of HD.

HTT lowering can be achieved in either an allele-selective manner, where only mHTT is reduced, or in a non-selective manner, where both alleles are suppressed indiscriminately. Both strategies offer strengths and potential pitfalls for therapeutic development with both allele-selective (ClinicalTrials.gov identifier: NCT03225833, NCT03225846) and non-selective (NCT03342053, NCT03761849) HTT lowering agents currently being evaluated in clinical trials for HD.

Allele-selectivity can be achieved by either targeting the expanded CAG tract on the mutant *HTT* allele (37–40) or by targeting other intragenic *HTT* polymorphisms, such as single nucleotide polymorphisms (SNP) and insertion/deletion variants that are in linkage disequilibrium with the HD mutation (28,41–45). Although allele-selective reduction of mHTT may represent a more direct therapeutic strategy for HD, this approach presents added technical challenges for drug development. For CAG targeted therapies, there is the potential to induce non-selective HTT suppression since the wild type *HTT* allele also contains a CAG tract and selectivity of CAG-targeted agents against the mutant *HTT* allele may be reduced at repeat lengths most common in the adult-onset HD range (37,46). Moreover, there is also the potential for inducing off-target suppression of other CAG tract containing genes, which are common in the genome (47). For polymorphism targeted allele-selective approaches, prioritizing targets that are closely linked with the HD mutation that would be the most useful for treating the greatest number of patients becomes an important consideration. This is due to *HTT* haplotype heterogeneity between HD populations of different ethnicities (41,48–50). As such, a drug designed to target a single polymorphism could offer an allele-selective treatment option for approximately 50% of patients, and increasing patient coverage beyond this would require additional drugs targeting other polymorphisms commonly associated with the HD mutation.

Non-selective HTT lowering offers a more straightforward therapeutic development path where a single drug could be used to treat the entire global HD population. However, it remains unclear what magnitude of long-term non-selective suppression will be tolerated given the important cellular roles of wtHTT (10,51,52). Preclinical studies in rodents and non-human primates have provided evidence that a transient, partial reduction of wtHTT may be safe and well tolerated. Results from the first HTT lowering phase I/IIa clinical trial (NCT02519036) evaluating ascending doses of a non-selective antisense oligonucleotide, IONIS-HTTRx (RG6042), administered monthly by intrathecal (IT) injection showed that the drug was relatively safe and well tolerated at all doses tested for up to 13 weeks (53). However, the duration and magnitude of wtHTT lowering that will be tolerated long term remains unclear.

HTT lowering agents can be further classified by their ability to induce either transient or long-term HTT suppression. Transient HTT suppression approaches require repeated re-administration of the therapeutic to maintain reduced levels of HTT. In contrast, long-term HTT suppression employs viral vectors to deliver expression cassettes that continuously produce the therapeutic agent following a single administration. Among viral vectors, adeno-associated virus (AAV) vectors are preferred for delivery of therapeutic agents since they provide long term transgene expression, they remain predominantly episomal following transduction (thus strongly reducing the potential of random insertional mutagenesis) and they do not exhibit pathogenicity in humans. Numerous AAV capsid serotypes have been identified that can alter cellular tropism, tissue distribution, transduction efficiency and transgene expression *in vivo* (54–58).

AAV serotype 5 (AAV5) has been shown to efficiently transduce neurons, astrocytes, microglia and ependymal cells of the CNS following direct intraparenchymal injection (57,59). We have previously reported the design and proof-of-concept, for short-term suppression of total human HTT using an AAV5 encoding a miRNA targeting exon 1 of human HTT (miHTT) in a humanized mouse model of HD, Hu128/21, which expresses two full length, human *HTT* transgenes heterozygous for the HD mutation on the *Htt*-/- background (60,61). Moreover, we have demonstrated tolerability of AAV5-miHTT and potent mHTT suppression in an acute rat model of HD (62) as well as in a large model of HD, the transgenic HD minipig (63). We have also shown that AAV5-miHTT treatment leads to survival and motor coordination improvements in the R6/2 mouse model of HD (64). In the current study, we evaluate tolerability, pharmacodynamics and efficacy of the same AAV5-miHTT in the Hu128/21 model of HD (61).

## MATERIALS AND METHODS

### Mice

Experiments were performed using Hu128/21 HD model mice and Hu21 control littermates (61). Animals were maintained under a 12 h light:12 h dark cycle in a clean barrier facility and given free access to food and water. Experiments were performed with the approval of the Animal Care Committee at the University of British Columbia (A16-030).

### Baseline behavioural assessment

Rotarod training was performed as in (61). Briefly, mice were trained over three 120-second trials per day over 3 days on a fixed speed 18 RPM rotarod. Mice that fell were immediately returned to the rod. The mean number of falls and latency to the first fall per day were scored for each mouse.

### Surgical AAV delivery

After completion of baseline behaviour testing, AAVs were delivered intracranially. Mice were anesthetized with isoflurane and placed into a stereotaxic frame. The scalp was prepared and an incision made along the mid-line. The skull was dried to enhance visibility of sutures and landmarks. A dental drill was used to make bilateral burr holes at 0.8 mm anterior and 1.8 mm lateral to Bregma. A Hamilton syringe with 30 gauge needle (Hamilton, Reno, NV) was loaded first with 3 μl of sterile saline and then with 2 μl of viral solution diluted to appropriate titre in sterile saline. The needle was lowered to 3.5 mm below the surface and the virus injected at 0.5 μl/min using an UltraMicroPump with Micro4 controller (World Precision Instruments). The needle was left in place for 5 min and then withdrawn slowly.

### Behaviour testing

Body weight and motor function were scored longitudinally at 2-month intervals from 2 to 8 months of age. Psychiatric behaviour was assessed at 3 and 9 months of age during exploration of a brightly lit open field. Cognitive function was assessed at 2 months of age during rotarod training as described above and at 9 months of age by object location and recognition learning assays.

Accelerating rotarod testing was performed as in (61). Briefly, mice were tested over three 300 s trials on a rotarod accelerating from 5 to 40 RPM. Mean latency to fall was scored for each mouse. Mice that gripped the rod without walking for three consecutive rotations were considered to have fallen.

Anxiety-like behavior was evaluated at 3 and 9 months of age during exploration of a brightly lit open field as in (61). Briefly, mice were placed in the lower left corner of a 50 × 50 cm open top box under bright lighting and allowed to explore for 10 min. Mice were recorded via ceiling-mounted video camera and total distance travelled, mean velocity, entries into the center of the open field, and total time spent in the center of the field were scored by Ethovision XT 7. Animals with <3 cm/s mean velocity were excluded from analysis for having failed to explore the field.

At 9 months of age testing was extended to include object learning assays as in (61). Briefly, at the conclusion of open field testing, mice were placed into their home cage for a 5 min inter-trial interval (ITI). Two objects were placed into the upper right corners of the box and the mice returned for a 5 min exploration. Investigations to each object were scored by Ethovision XT and the percentage of investigation to the object on the right calculated. The mice were then removed for a 5 min ITI and the object on the right was moved to the lower right corner. Mice were given another 5 min exploration and percentage of investigations to the target object on the right was scored. Preference for investigating the object in the novel location was evaluated as a measure of spatial learning. For the novel object location learning assay, mice who failed to explore both objects and trials in which tracking failed >10% of the time were excluded from analysis.

### Tissue collection and processing

For mice allotted for terminal molecular and biochemical analysis, brains were removed and placed on ice for 1 min to increase tissue rigidity. Brains were then microdissected by region. Striata and cortices were preserved in RNAlater (Ambion) overnight at 4°C and then stored at −80°C until use. Hippocampi, cerebella, and the rest of the brain were flash frozen in liquid nitrogen and stored at −80°C until use.

Mice allotted for terminal histological analysis were perfused transcardially with phosphate buffered saline (PBS) and 4% paraformaldehyde (PFA). Brains were removed and post-fixed in 4% PFA in PBS for 24 h at 4°C. The following day brains were cryoprotected in 30% sucrose with 0.01% sodium azide. Once equilibrated, brains were divided into forebrain and cerebellum, and forebrains were frozen on dry ice, mounted in Tissue-TEK O.C.T. embedding compound (Sakura), and cut via cryostat (Leica CM3050S) into a series of 25 μm coronal sections free- floating in PBS with 0.01% sodium azide.

### Neuropathology and brain histology

For evaluation of viral distribution, a series of sections spaced 200 μm apart and spanning the striatum were co-stained with primary rabbit anti-GFP (1:1000, Life Technologies) and mouse/rat anti-DARPP-32 (1:1000, R&D Systems MAB4230) and secondary goat anti-rabbit (1:500, Alexa-fluor 488) and goat anti-rat (1:500, Alexa-fluor 568) secondary antibody. Sections were mounted using ProLong Gold Antifade mounting reagent (ThermoFisher P36930) with DAPI (Life Technologies). Sections were imaged with a 2.5× objective (Zeiss) using a Zeiss Axioplan 2 microscope and Coolsnap HQ Digital CCD camera (Photometrics, Tucson, AZ, USA) and MetaMorph software (Molecular Devices).

Stereological volumetric analysis was performed as in (61). Briefly, a series of sections spaced 200 μm apart and spanning the striatum were stained for NeuN (1:1000, Millipore MAB377) using biotinylated anti-mouse secondary antibody (1:1000, Vector Laboratories), the ABC Elite Kit (Vector) to amplify signal and 3,3′-diaminobenzidine (DAB, Thermo Scientific) detection. Structures were traced using Stereo Investigator software (MBF Bioscience) and volumes determined using the Cavalieri principle. Striatal, cortical and corpus callosum volumes were measured at 9 months of age. Lateral ventricle size was measured at 4 months of age.

Striatal DARPP-32 immunoreactivity was evaluated as in (61). Briefly, a series of four mid-striatal sections were stained for DARPP-32 (1:500, R&D Systems MAB4230) using biotinylated anti-rat secondary antibody (1:1000, Vector Laboratories), the ABC Elite Kit (Vector) to amplify signal, and 3,3′-diaminobenzidine (DAB, Thermo Scientific) detection. Sections were imaged as above with a 5× objective (Zeiss). Integrated optical density (IOD) of DARPP-32 staining was calculated using Fiji ImageJ software (65).

To evaluate neuroinflammation and viral tropism in treated mice, free floating sections were blocked for 0.5 h at RT in and 5% normal goat or normal donkey serum with 0.1% Triton X-100 in PBS. Primary antibodies were incubated overnight at RT and secondary antibodies were incubated for 2 h at RT. For neuroinflammation, a series of sections spaced 200 μm apart and spanning the striatum were co-stained with primary rabbit anti-GFP (1:1000, Life Technologies) and primary goat anti-Iba1 (1:1000, Abcam ab107159) and secondary donkey anti-rabbit (1:500, Alexa-fluor 488) and donkey anti-goat (1:500, Alexa-fluor 594) secondary antibody. Another set of sections were co stained with primary rabbit anti-GFP and secondary goat anti-rabbit (1:500, Alexa-fluor 488) and mouse anti-GFAP CY3 conjugate (1:500, Sigma C9205). These sections were also used to evaluate viral tropism in addition to sections co-stained with primary rabbit anti-GFP (1:1000, Life Technologies) and either mouse anti-NeuN (1:1000, Millipore MAB377) or mouse/rat anti-DARPP-32 (1:1000, R&D Systems MAB4230) with secondary goat anti-rabbit (1:500, Alexa-fluor 488) and secondary goat anti-mouse (1:500, Alexa-fluor 568). Sections were mounted using ProLong Gold antifade mounting reagent with DAPI (Life Technologies). For qualitative analysis of neuroinflammation and co-localization, sections were imaged with 5× and 20× objectives (Zeiss) using the microscope described above.

For semi-quantitative analysis of co-localization, GFP/NeuN, GFP/GFAP, GFP/Iba1 and GFP/DARPP-32 stained sections were imaged with a 40× objective using a Leica SP8 STED confocal microscope. Image format was 512 × 512 pixels at a scan speed of 400 Hz. A series of eight images per animal were taken for analysis. Region of interest within the striatum was chosen randomly. All eight images for each of the animals were taken with same laser and gain setting to allow comparison.

Colocalization analysis was performed using Fiji ImageJ software (65). Images were imported using the bio-formats plug-in and colour channels were separated. Background on each channel was subtracted using the rolling ball method with a ball radius of 10–50 pixels. A threshold was then applied to either NeuN, GFAP, Iba1 or DARPP-32 to highlight each cell type, respectively. Particle analysis was then performed on the thresholded image using a particle size (micron^2^) of 10-infinity. Regions of interest were then applied to the GFP channel and IOD was measured. IODs with values >1500 in the GFP channel were used to define overlap. Colocalization using this method was validated manually on two images per animal.

### HTT quantitation

Full length wtHTT and mHTT protein was quantified in dissected tissues by allelic separation immunoblotting as in (43). Briefly, 40 μg of total protein was resolved on 10% low-BIS acrylamide gels, transferred to 0.45 μm nitrocellulose and probed for total HTT (MAB2166, Millipore) and calnexin (Sigma C4731) as a loading control. Primary antibodies were detected with IR dye 800CW goat anti-mouse (Rockland 610-131-007) and AlexaFluor 680 goat anti-rabbit (Molecular Probes A21076)-labelled secondary antibodies, and the LiCor Odyssey Infrared Imaging system. Densitometry on band intensities was performed using the LiCor Image Studio Lite software, and HTT intensities were normalized to calnexin loading control and then to the same allele for saline treated mice on the same membrane.

### Vector genome copies quantitation

DNA isolation from the cortices of Hu21 and Hu128/21 mice at 7 months post-injection was performed using the DNeasy^®^ Blood and Tissue kit protocol (Qiagen). DNA was eluted in 50μL H_2_O and the concentration measured by using the NanoDrop2000 (Thermo Fisher Scientific). After determining the concentration, genome copy levels were determined by a TaqMan qPCR assay using forward primer: CCCACCAGCCTTGTCCTAAT, reverse primer: GTTCCTCAGATCAGCTTGCAT and probe: ACGGGCCCGTCGACTGCAGAGGCC (Applied Biosystems catalog # 4316032).

### Mature miHTT quantitation

RNA isolation from the cortices of Hu21 and Hu128/21 mice at 7 months post-injection was performed using the Direct-zol™ RNA MiniPrep kit (Zymo Research). RNA concentrations were measured using the NanoDrop2000 (Thermo Fisher Scientific). To determine miHTT microRNA molecule levels, the TaqMan® MicroRNA Reverse Transcription (RT) Kit (Thermo Fisher Scientific) and gene specific RT primers to target the miHTT mature guide strand (Thermo Fisher Scientific) were used. A reverse transcription control was included in the reaction. Mature miHTT molecules were quantified by performing a RT-PCR and TaqMan qPCR specifically developed to only reverse transcribe and measure AAV5-miHTT in combination with an AAV5-miHTT standard line. RT-PCRs were performed on a Biometra GMBHT advanced Thermocycler (Biometra GmbH). The RNA standard line consisting of the miHTT mature guide strand sequence (miRNA standard line) was reverse transcribed from a mature guide miHTT RNA sequence using the same cDNA synthesis method as described above. A miRNA standard line ranging from 100 pg to 1 fg was included on each qPCR plate used to determine the miHTT molecules per reaction by interpolating from the miRNA standard line. The concentration of miHTT was calculated as pmol per gram of RNA input.

### Statistical analysis

In all figures, data are presented as means ± SEM. All statistics were performed with the software GraphPad Prism v.8 (GraphPad) with the exception of Supplementary Figures S8 and S9 which were performed using Stata IMP v.15 software (Stata Corp. 2019; College Station, TX, USA). A *P*-value of <0.05 was considered significant for all analyses.

Statistical analyses of AAV5-miHTT dose effect on viral vector genome copies and concentration of mature miRNA molecules in the cortex of Hu21 or Hu128/21 were performed using one-way ANOVA with posthoc analysis using Tukey's multiple comparison test. Linear regression analysis was conducted on these data to explore the relationship between either dose and viral genomes copies or dose and pmol miHTT/g RNA in the cortex. We scaled the dose using 0 for saline control, 1 for 5.20E+09 (low), 2 for 2.60E+10 (medium) and 3 for 1.30E+11 (high). Fitting was done to individual replicates.

Statistical analysis of HTT suppression in Hu128/21 mice was done using two-way ANOVA with posthoc analysis using the Bonferroni multiple comparison test. With this data set, we evaluated three hypotheses: (i) Was there a significant effect of AAV5-miHTT dose on HTT suppression at each time point in either the striatum or cortex? (ii) Was there a treatment duration effect on HTT in the striatum and cortex? (iii) Was there significant difference in HTT suppression with AAV5-miHTT in the striatum compared to cortex? Statistical analysis of HTT suppression in Hu21 mice was done using one-way ANOVA with posthoc analysis using the Tukey's multiple comparison test. With this data set we evaluated whether there was a significant effect of AAV5-miHTT dose on HTT suppression in either the striatum or cortex.

Statistical analyses of the longitudinal accelerating rotarod and open field tests in Hu21 and Hu128/21 mice were performed using a mixed effects model. Posthoc analyses were performed using Tukey's or Sidak's multiple comparison tests. In these data sets, we evaluated two primary hypotheses: (i) Was there a significant difference between the saline treated Hu128/21 group and the saline-treated Hu21 group at multiple time points? (ii) Was there a significant AAV5-miHTT treatment versus saline effect in either the Hu21 or Hu128/21 groups at multiple time points? Statistical analysis of the novel object location test in both Hu21 or the Hu128/21 mice was performed using unpaired, one-tailed *t*-test to compare the means of trials 1 and 2.

Statistical analyses of stereological measurements (striatum, cortex and corpus callosum volume) in Hu21 and Hu128/21 were performed using both (i) unpaired, two-tailed t-test to test if there was a significant difference between the saline treated Hu128/21 group and the saline-treated Hu21 group and (ii) one-way ANOVA to test if there was significant effect of AAV5-miHTT treatment versus saline effect in the Hu21 or Hu128/21 groups.

Statistical analysis of lateral ventricle size in either Hu21 or Hu128/21 was performed using one-way ANOVA with posthoc analysis using the Tukey's multiple comparison test. Statistical analysis of longitudinal body weight in Hu21 and Hu128/21 mice was performed using mixed-effects model. Posthoc analyses was performed using Tukey's multiple comparison tests. Comparison of survival curves in either Hu21 or Hu128/21 treated groups was performed using the log-rank (Mantel-Cox) test.

For Supplementary Figures S8 and S9, one-way ANOVA test was used to compare the striatal volume or corpus callosum volume in response to different doses of viral genomes for Hu21 and Hu128/21 separately. Multiple linear regression analysis was conducted to explore the relationship between striatal volume or corpus callosum volume and dose of viral genomes, as well as genotype. In the regression analysis, we defined either striatal volume or corpus callosum volume as a dependent variable while dose of viral genomes and genotype were defined as independent predictors. We scaled the dose of viral genomes as 0 for control, 1 for 5.20E+09, 2 for 2.60E+10 and 3 for 1.30E+11. In addition, we coded genotype Hu21 as 1, and Hu128/21 as 2. As we noticed that the striatal volume of Hu21 tended to be greater than that of Hu128/21, and Hu21 striatal volume decreased with increasing dose whereas Hu128/21 striatal volume increased with increasing dose, we decided to add an interactive item between dose and genotype to the regression model to reflect such trends. In analysis of the relationship between corpus callosum volume and dose of viral genomes and genotype, we also included a quadratic item for dose of viral genomes since we observed that with increasing AAV5-miHTT dose, corpus callosum volume initially increased, and then decreased. All the statistical tests were two-tailed with significance level set at α < 0.05.

## RESULTS

### Study design overview

**Figure 1.**
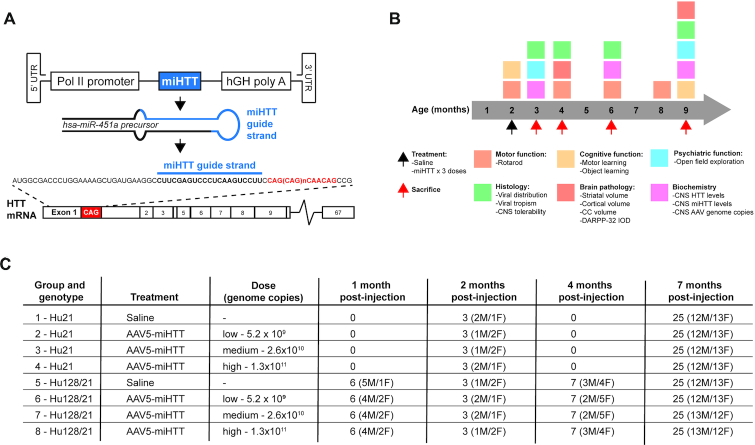
Overview of study design. (**A**) Cartoon representation of miHTT expression cassette and target sequence within exon 1 of human *HTT*. (**B**) Overview of study design highlighting timeline of AAV5-miHTT injections and endpoints. (**C**) Overview of cohort design with breakdown of Hu21 and Hu128/21 cohort animal numbers and sex distribution for each of the harvest timepoints.

Hu128/21 and control Hu21 animals received bilateral intrastriatal infusions by convection-enhanced delivery (CED) of either saline or three ascending doses of AAV5-miHTT (low: 5.2 × 10^9^, medium: 2.6 × 10^10^, or high: 1.3 × 10^11^ genome copies per mouse) at 2 months of age and were evaluated until 9 months of age. AAV5-miHTT targets a sequence upstream of the CAG tract within exon 1 of the human *HTT* transcript (Figure 1A). Viral distribution was assessed by immunohistochemistry (IHC) at 3, 6 and 9 months of age (Figure 1B). Viral tropism and tolerability was evaluated by IHC at 4 months of age (Figure 1B). Target engagement was assessed by quantification of HTT levels using western blot at 3, 6 and 9 months of age (Figure 1B). Behavioural assessments of motor performance were evaluated using the accelerating rotarod test at 2, 4, 6 and 8 months of age; psychiatric-like phenotypes were evaluated using the open field exploration test at 3 and 9 months of age; and cognitive measures were assessed using the novel object location test at 9 months of age (Figure 1B). Neuropathology was evaluated using IHC and stereology at 9 months of age (Figure 1B). Animal numbers and sex distribution for each treatment group and collection timepoint are outlined in Figure 1C. All biochemical, behavioural and neuropathological data from this study are summarized in Supplementary Table S1.

### Broad distribution and tropism of AAV5 in Hu128/21 brain

**Figure 2.**
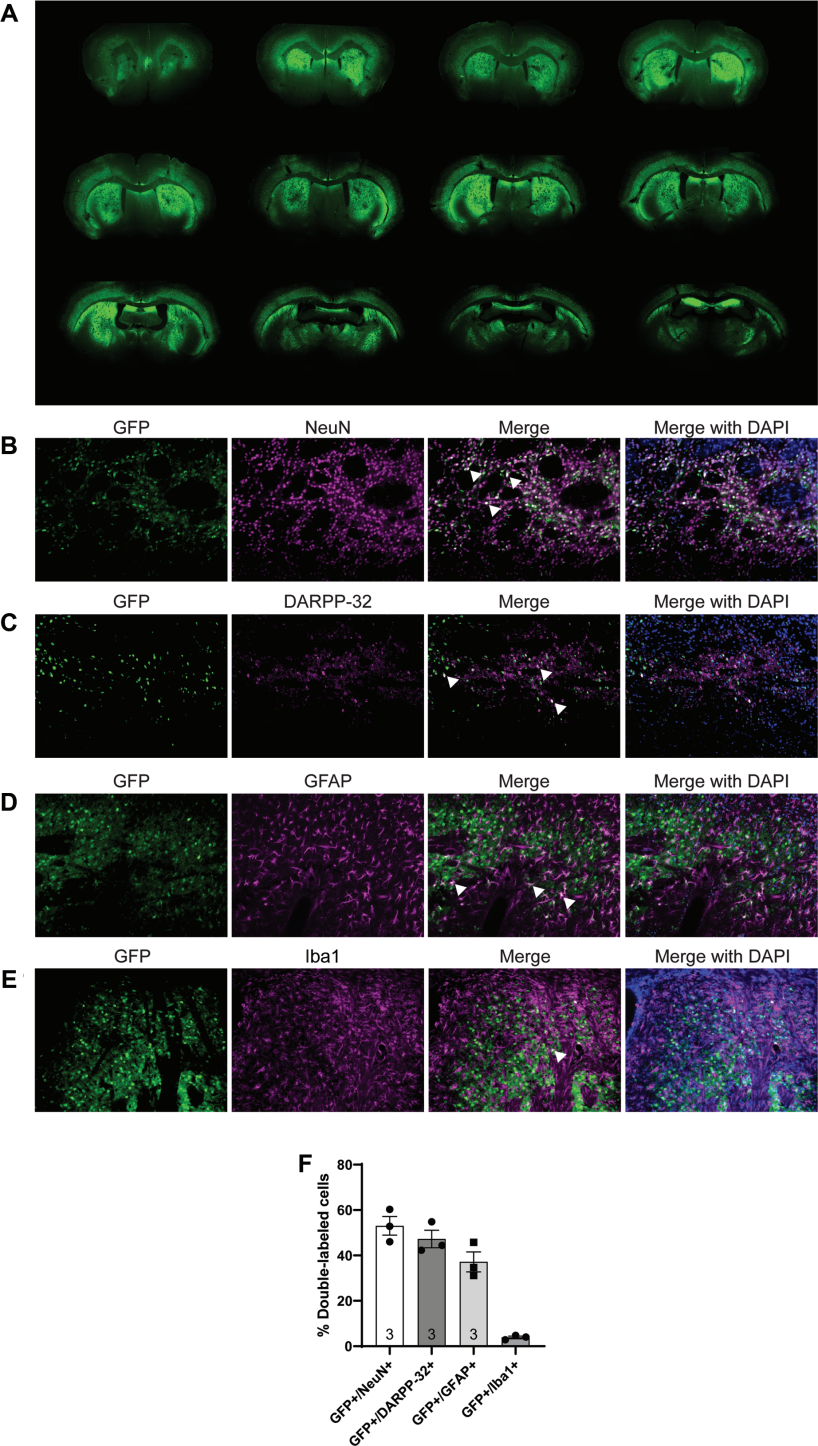
Widespread distribution and broad cell-type tropism of AAV5-GFP following bilateral intrastriatal injection in the Hu128/21 humanized model of HD. (**A**) A series of sections spanning the striatum was stained for GFP to evaluate viral distribution at 9 months of age (7 months post-injection). Tropism was evaluated qualitatively by co-localization of GFP from AAV-miScr treated animals with (**B**) NeuN, a general marker for neurons, (**C**) DARPP-32, a marker for medium spiny neurons (MSNs), (**D**) GFAP, a marker for astrocytes and (**E**) Iba1, a marker for microglia. (**F**) Quantification of the % overlap between GFP+ and either NeuN+, GFAP+, Iba1+ and DARPP-32+ cells in the striatum of AAV-miScr treated animals.

To evaluate the extent of AAV5 distribution in the CNS, Hu128/21 mice received bilateral intrastriatal infusions by CED at 2 months of age with an AAV5 vector expressing green fluorescent protein (AAV5-GFP) and were harvested for IHC. AAV5-GFP showed broad distribution at all time points evaluated and could clearly be observed in the striatum (site of injection), the hippocampus and deeper layers of the cortex at 7 months post-injection (Figure 2A). To examine cellular tropism of AAV5 within the brain, Hu128/21 received bilateral intrastriatal injections with a scramble miRNA tagged with GFP (AAV5-miScr-GFP) and were harvested for IHC at 2 months post-injection. We qualitatively observed co-localization of GFP with NeuN (Figure 2B), DARPP-32 (Figure 2C), GFAP (Figure 2D) and Iba1 (Figure 2E) demonstrating transduction of neurons, striatal medium spiny neurons, astrocytes and microglia, respectively. Semi-quantitative co-localization analysis demonstrates that AAV5-miScr-GFP transduces 53.06 ± 7.14% (mean ± S.D.) of NeuN+ neurons, 47.22 ± 6.71% of DARPP-32 neurons, 37.20 ± 7.62% of GFAP+ astrocytes and neural progenitors, and 3.88 ± 0.97% of Iba1+ microglia within the striatum. This data highlights the broad cellular tropism of AAV5, consistent with previous reports for this serotype (57,59). We also observed that both AAV5-GFP and AAV5-miScr-GFP induced robust astrogliosis (Supplementary Figure S1) and microgliosis (Supplementary Figure S2), abnormal striatal morphology (Supplementary Figure S3) and increased lateral ventricle size (Supplementary Figure S4, Hu21 treated groups: One-way ANOVA *P* < 0.0001. Hu128/21 treated groups: One-way ANOVA *P* = 0.0036) in both Hu21 and Hu128/21 mice. Due to the poor tolerability of GFP transgenes, AAV5-GFP and AAV5-miScr-GFP were excluded from the efficacy study, and saline vehicle was used as a control.

### AAV5-miHTT is tolerated in Hu21 and Hu128/21 mice

We next evaluated the tolerability of AAV5-miHTT by performing IHC for markers of astrogliosis (GFAP) and microgliosis (Iba1) in the brains of Hu21 and Hu128/21 animals at 2 months post-injection. These markers have been used previously to qualitatively evaluate glial activation as a measure of overt tolerability (45). We observed that AAV5-miHTT high dose induced astrogliosis in both Hu21 and Hu128/21 brain, whereas this was absent from mice injected with AAV5-miHTT at medium and low doses (Supplementary Figure S1). In contrast, AAV5-miHTT did not induce any microgliosis (Supplementary Figure S2), abnormal striatal morphology (Supplementary Figure S3) or lateral ventricle size changes (Supplementary Figure S4) at any dose tested. Body weight was not altered following treatment with any dose of AAV5-miHTT compared to saline in Hu21 (Supplementary Figure S5A, Mixed-effects model treatment *P* = 0.4077, age *P* < 0.0001, treatment × age *P* = 0.6130) or Hu128/21 mice (Supplementary Figure S5B, Mixed-effects model treatment *P* = 0.0997, age *P* < 0.0001, treatment × age *P* = 0.1509) at 2, 4, 6 or 8 months of age. Finally, there was no effect of AAV5-miHTT on survival in either Hu21 (Supplementary Figure S5C, Log-rank test: Chi square = 0.0005084, df = 3, *P* > 0.9999) or Hu128/21 mice (Supplementary Figure S5D, Log-rank test: Chi square = 3.056, df = 3, *P* = 0.3831) up to 7 months post-injection. These results suggest that AAV5-miHTT was tolerated in both Hu21 and Hu128/21 mice for up to 7 months but high dose AAV5-miHTT treatment induced astrogliosis.

### Efficient transduction and potent suppression of HTT in Hu128/21 mice with AAV5-miHTT

**Figure 3.**
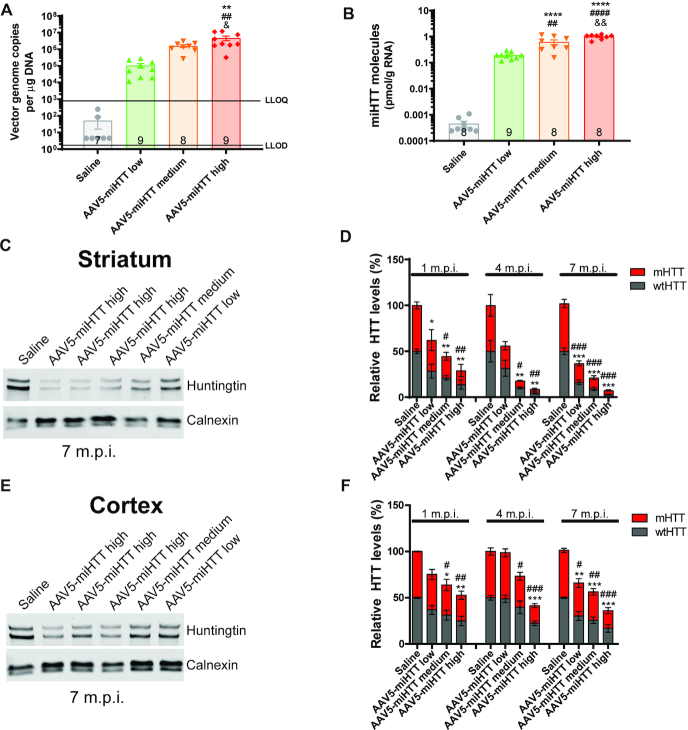
AAV5-miHTT shows potent and sustained suppression of total HTT in Hu128/21 mice. (**A**) AAV5-miHTT viral vector genome copies in the cortex of Hu128/21 at 7 months post-injection (one way ANOVA *P* = 0.0007, Tukey's multiple comparison test **P = 0.0021 high dose compared to saline, ^##^*P* = 0.0013 high compared to low dose, ^&^*P* = 0.0446 high compared to medium dose. *N* = 7–9). LLOQ: Lower limit of quantification, LLOD: lower limit of detection. (**B**) Total picomoles of mature miHTT miRNA per gram of total RNA in the cortex of Hu128/21 mice at 7 months post-injection (one way ANOVA *P*< 0.0001, Tukey's multiple comparison test ****P< 0.0001 medium and high dose compared to saline, ^##^*P* = 0.012 medium and ^####^*P*<0.0001 high compared to low dose, ^&&^*P* = 0.0047 high compared to medium dose. *N* = 7–9). Representative western blot showing dose-dependent HTT lowering in (**C**) striatum and (**E**) cortex at 7 months post-injection. Quantification of wt and muHTT levels in the (**D**) striatum (* for wtHTT and ^#^ for mHTT. **1 month post-injection** two-way ANOVA dose *P* = 0.0002, HTT *P* = 0.6134, interaction *P* = 0.9767. Bonferroni post-tests: * *P*<0.05 low dose, ^#^*P*<0.05 and **P<0.01 medium dose, ** and ^##^*P*< 0.01 high dose compared to saline, *N* = 3. **4 months post-injection** two way ANOVA dose *P*<0.0001, HTT *P* = 0.6882, interaction *P* = 0.9805. Bonferroni post-tests **P< 0.01 and ^#^*P*< 0.05 medium dose, **P< 0.01 and ^##^*P*< 0.01 high dose compared to saline, *N* = 4–6. **7 months post-injection** two-way ANOVA dose *P*< 0.0001, HTT *P* = 0.1712, interaction *P* = 0.8258. Bonferroni post-tests *** and ^###^*P*< 0.001 low, medium and high dose compared to saline, *N* = 6–9) and (**F**) cortex (* for wtHTT and ^#^ for mHTT, **1 month post-injection** two-way ANOVA dose *P* = 0.0006, HTT *P* = 0.6582, interaction *P* = 0.9914. Bonferroni post ^#^ and * *P*< 0.05 medium dose, ** and ^##^*P*< 0.01 high dose compared to saline, *N* = 3. **4 months post-injection** two-way ANOVA dose *P*< 0.0001, HTT *P* = 0.4490, interaction *P* = 0.8036. Bonferroni post-tests ^#^*P*<0.05 medium dose, *** and ^###^*P*< 0.001 high dose compared to saline, *N* = 4–6. **7 months post-injection** two-way ANOVA dose *P*< 0.0001, HTT *P* = 0.2641, interaction *P* = 0.8874. Bonferroni post-tests **P< 0.01 and ^#^*P*< 0.05 low dose, ***P< 0.001 and ^##^*P*< 0.01 medium dose, *** and ^##^*P*< 0.001 high dose compared to saline, *N* = 6–9) at 1, 4 and 7 months post-injection. M.P.I. = months post-injection.

To evaluate transduction efficiency of AAV5-miHTT in Hu128/21 mice, concentrations of viral vector DNA and mature miHTT miRNA molecules were evaluated in the cortex at 7 months post-injection. A significant dose dependent increase in vector DNA levels, up to 4.8 × 10^6^ genome copies per μg of genomic DNA with high dose treatment, was observed in the cortex of AAV5-miHTT treated animals. (Figure 3A, one-way ANOVA *P* = 0.0007. Linear regression of dose vs genome copies in Hu128/21 treated groups *r*^2^ = 0.3746, non-linear slope *P* = 0.0002). Moreover, a significant dose-dependent increase in mature miHTT miRNA molecules, up to 1.003 pmol/g of total RNA, were measured in the cortex of Hu128/21 mice at 7 months post-injection (Figure 3B, one way ANOVA *P* < 0.0001. Linear regression of dose vs pmol miHTT/g RNA in Hu128/21 treated groups *r*^2^ = 0.7785, non-linear slope *P* < 0.0001). Since both vector DNA and the miHTT miRNA molecules were detected in the cortex, adjacent to the site of AAV5-miHTT injection in the striatum, this may suggest that AAV5 undergoes active axonal transport via cortico-striatal pathways (66).

To evaluate the pharmacodynamic properties and HTT target engagement following AAV5-miHTT treatment in Hu128/21 mice, levels of soluble, full-length human wild type (wt) and mutant HTT (mHTT) protein were quantified in both the striatum and cortex at 1, 4 and 7 months post-injection. We have previously found that levels of HTT mRNA do not always accurately reflect changes at the protein level (43,45), therefore we chose to measure levels of HTT protein since we believe this to be more informative for assessing target engagement of a HTT lowering therapy. All raw Western blots used for quantification of HTT target engagement can be found in Supplementary Figure S6.

We observed a dose-dependent reduction of full length HTT (both wtHTT and mHTT) in the striatum at each timepoint with low, medium and high dose treatment resulting in 63%, 78%, and 92% suppression at 7 months post-injection, respectively (Figure 3C and D, **1 month post-injection** two-way ANOVA dose *P* = 0.0002, HTT *P* = 0.6134, interaction *P* = 0.9767, **4 months post-injection** two-way ANOVA dose *P* < 0.0001, HTT *P* = 0.6882, interaction *P* = 0.9805, **7 months post-injection** Two-way ANOVA dose *P*<0.0001, HTT *P* = 0.1712, interaction *P* = 0.8258).

Moreover, there was also a dose-dependent reduction of full length HTT in the cortex at each timepoint post-injection with low, medium and high dose treatments resulting in 34%, 43% and 64% HTT suppression at 7 months post-injection, respectively (Figure 3E and F, **1 month post-injection** Two-way ANOVA dose *P* = 0.0006, HTT *P* = 0.6582, interaction *P* = 0.9914, **4 months post-injection** Two-way ANOVA dose *P* < 0.0001, HTT *P* = 0.4488, interaction *P* = 0.8040, **7 months post-injection** two-way ANOVA dose *P* < 0.0001, HTT *P* = 0.2641, interaction *P* = 0.8874). Consistent with a non-selective HTT lowering miRNA, we did not observe a significant difference between wt and mHTT suppression with AAV5-miHTT at any timepoint or brain region.

A trend towards a treatment duration-dependent reduction in wt and mHTT levels with AAV5-miHTT treatment was observed in the striatum but this did not reach significance (**wtHTT**: two-way ANOVA timepoint *P* = 0.0827, treatment *P* < 0.0001, interaction 0.6555, **mHTT**: two-way ANOVA timepoint *P* = 0.0868, treatment *P* < 0.0001, interaction 0.8858). We did however observe a significant treatment duration-dependent suppression of wtHTT (two-way ANOVA time point *P* = 0.0073, treatment *P* < 0.0001, interaction 0.3332. Bonferroni post-test 4 versus 7 months post-injection for low dose *P* < 0.05) but not mHTT in the cortex (two-way ANOVA timepoint *P* = 0.2606, treatment *P* < 0.0001, interaction 0.4154).

Consistent with intrastriatal delivery of AAV5-miHTT, we observed a significantly higher magnitude of both wt and mHTT lowering in the striatum compared to the cortex at 7 months post-injection (**wtHTT**: two-way ANOVA brain region *P* < 0.0001, dose *P* < 0.0001, interaction *P* = 0.1331. **mHTT**: two-way ANOVA brain region *P* < 0.0001, dose *P* < 0.0001, interaction *P* = 0.0631).

### AAV5-miHTT may ameliorate cognitive and psychiatric, but not motor phenotypes in Hu128/21 mice

We next evaluated efficacy of AAV5-miHTT treatment on improving motor, cognitive and psychiatric-like behavioral phenotypes in Hu128/21 mice. The accelerating rotarod is a well described test to evaluate motor deficits in rodent models (67) and deficits in this test have been described longitudinally in Hu21 and Hu128/21 mice, with greater deficits in Hu128/21 mice yielding a genotypic difference (61). Consistent with previous reports, we observed a significant motor performance deficit as measured by latency to fall from the rotarod in Hu128/21 compared to Hu21 saline treated mice (Supplementary Figure S7, Mixed-effects model of Hu21 and Hu128/21 saline groups at all timepoints: genotype *P* = 0.0161, timepoint *P* < 0.0001, genotype × timepoint *P* = 0.1839). However, no effect of AAV5-miHTT treatment was observed on latency to fall in Hu128/21 mice up to 6 months post-injection (mixed-effects model of all Hu128/21 treated groups at all timepoints: treatment *P* = 0.5549, timepoint *P* < 0.0001, treatment × timepoint *P* = 0.8614). However, considering the obesity observed in both genotypes of this line of mice (61), they are not ideal for the assessment of motor phenotypes or therapeutic changes in motor performance.

**Figure 4.**
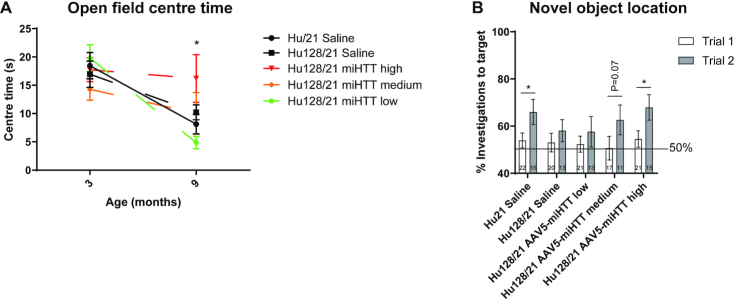
AAV5-miHTT may ameliorate psychiatric and cognitive phenotypes in Hu128/21 mice. (**A**) Open field centre time at 1 and 7 months post-injection (mixed-effects model on Hu21 and Hu128/21 saline treated groups at both time points; genotype *P* = 0.9760, timepoint *P*< 0.0001, genotype × timepoint *P* = 0.4023; mixed-effects model on all Hu128/21 treated groups at both timepoints; treatment *P* = 0.2820, timepoint *P*<0.0001, treatment x timepoint *P* = 0.0365. Tukey multiple comparison test: Hu128/21 low versus high dose *P = 0.0227, Hu128/21 miHTT high versus Hu128/21 saline = not significant. 3 months: *N* = 26–32, 9 months: *N* = 18–25). (**B**) Novel object location test at 9 months of age comparing Hu21 saline to Hu128/21 treated groups (unpaired *t*-test of trial 1 versus trial 2 for Hu21 saline *P = 0.0227, medium dose *P* = 0.0742 and high dose * *P* = 0.0182. *N* = 10–15).

Open field exploration is an established test used to measure exploratory activity and anxiety-like behaviour in rodents. Hu128/21 mice develop an anxiety-like phenotype from 3 months of age where they spend less time in the centre of the field than Hu21 control mice (61). However, in this study, we observed an anxiety-like phenotype in both Hu128/21 and Hu21 saline treated mice with both genotypes spending significantly less time in the centre of the field at 9 compared to 3 months of age (Figure 4A, Mixed effects model on Hu21 and Hu128/21 saline treated groups at both time points; genotype *P* = 0.9760, time *P* < 0.0001, genotype × time *P* = 0.4023). The observed anxiety in Hu21 saline treated control mice limits our ability to draw conclusions about the effects of AAV5-miHTT in Hu128/21 mice. There was a trend towards an increase in time spent in the centre of the field for Hu128/21 mice as a result of treatment but this failed to reach significance (Mixed-effects model for all Hu128/21 treated groups at both timepoints; treatment *P* = 0.2820, time *P* < 0.0001, treatment × time *P* = 0.0365). We did however observe a significant effect of AAV5-miHTT at 9 months of age with high dose treatment increasing time spent in the centre of the open field compared to low dose but not saline (Tukey multiple comparison test: Hu128/21 low vs high dose *P = 0.0227, Hu128/21 miHTT high versus Hu128/21 saline = not significant). Moreover, treatment with medium and high dose AAV5-miHTT in Hu128/21 mice blunted the progressive anxiety-like phenotype and maintained high centre time from 3 to 9 months.

The novel object location test measures spatial learning with wild type mice showing a preference for a known object in a novel location. Hu128/21 mice have a spatial learning deficit and show no preference in object location from 6 months of age (61). In this study, we observed a significant preference for the target object (the one that had been moved) in saline treated Hu21 but not Hu128/21 mice at 7 months post-injection (Figure 4B, Unpaired *t*-test on Hu21 saline treated group *P* = 0.0227 and Hu128/21 saline treated group *P* = 0.2114), recapitulating the expected spatial learning deficit of Hu128/21 mice. Treatment with AAV5-miHTT restored the preference for a known object in a novel location in a dose-dependent manner (Figure 4B, Unpaired *t*-test on Hu128/21 AAV5-miHTT treated groups *P* = 0.0182, *P* = 0.0742, and *P* = 0.2152 for high, medium, and low dose treated groups, respectively). This indicates that AAV5-miHTT can prevent onset of spatial learning deficits in Hu128/21 mice.

### AAV5-miHTT may prevent striatal volume loss and normalize striatal DARPP-32 levels in Hu128/21 mice

To further evaluate the efficacy AAV5-miHTT treatment, we assessed striatal, cortical and corpus callosum volumetric changes as well as DARPP-32 immunoreactivity in Hu128/21 mice at 7 months post-injection.

**Figure 5.**
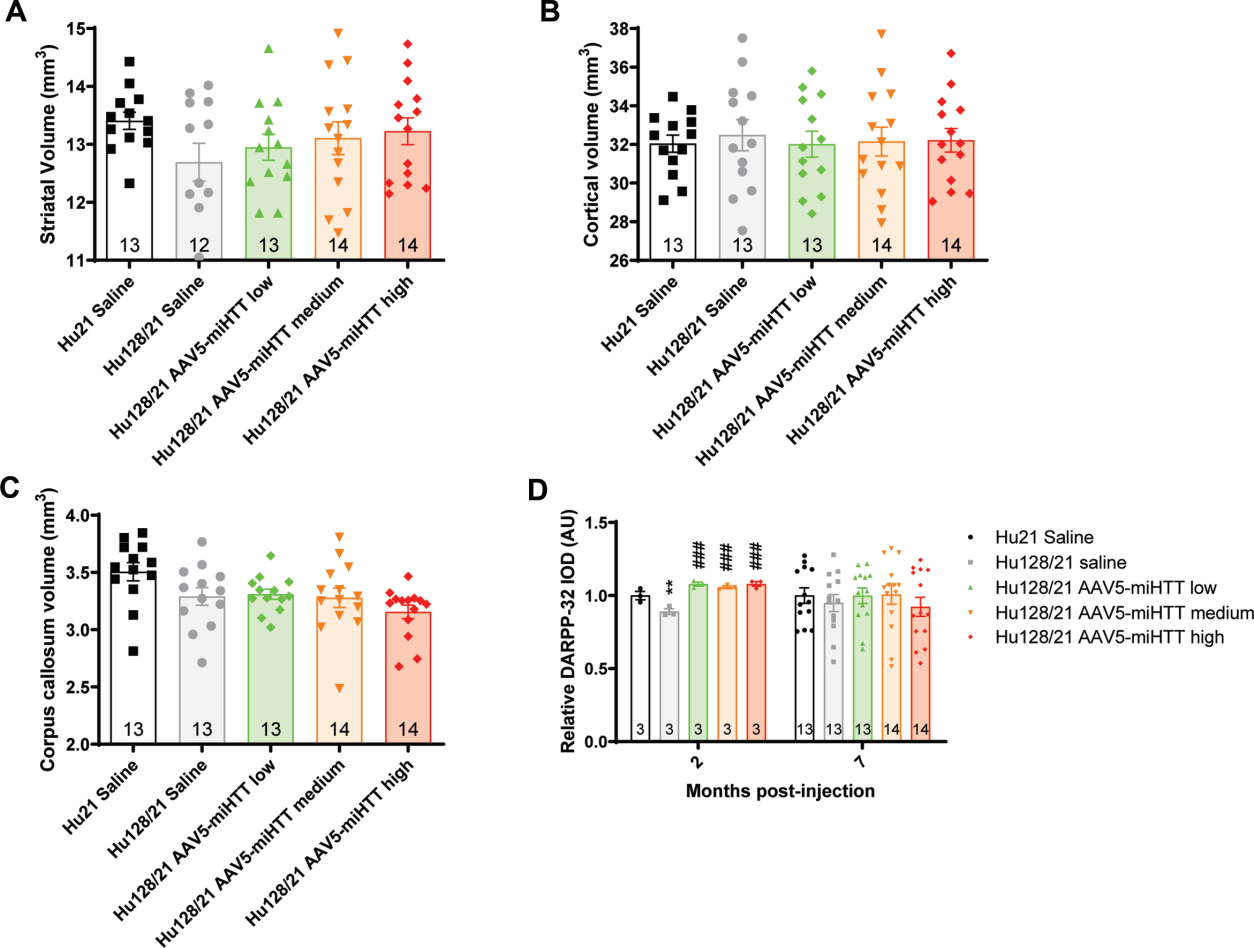
AAV5-miHTT may prevent striatal volume loss and normalize striatal DARPP-32 levels in Hu128/21 mice. Stereological evaluation of (**A**) striatal (unpaired *t*-test between Hu21 and Hu128/21 saline treated *P* = 0.0517; one-way ANOVA on Hu128/21 treated animals *P* = 0.465. *N* = 12–14), (**B**) cortical (unpaired *t*-test between Hu21 and Hu128/21 saline treated *P* = 0.6380; one-way ANOVA on Hu128/21 treated animals *P* = 0.9741. *N* = 13–14) and (**C**) corpus callosum volumes (unpaired *t*-test between Hu21 and Hu128/21 saline treated *P* = 0.0623; one-way ANOVA on Hu128/21 treated animals *P* = 0.3810. *N* = 13–14) at 7 months post-injection. (**D**) DARPP-32 immunoreactivity was evaluated by integrated optical density (IOD) in a series of 4 mid-striatal sections at 2 months post-injection (unpaired *t*-test between Hu21 and Hu128/21 saline treated **P = 0.0316; one-way ANOVA on Hu128/21 treated animals *P* = 0.0002. Tukey multiple comparison test ^###^*P*< 0.001 Hu128/21 saline versus high, medium and low doses. *N* = 3) and 7 months post-injection (unpaired *t*-test between Hu21 and Hu128/21 saline treated *P* = 0.5222; one-way ANOVA on Hu128/21 treated animals *P* = 0.7315. *N* = 13–14). Values were normalized to the mean value for saline treated Hu21 animals at each timepoint.

Striatal atrophy is a hallmark feature of HD that is recapitulated in Hu128/21 mice as early as 3 months of age (61). In this study, there was a strong trend towards a reduction in striatal volume in saline treated Hu128/21 compared to Hu21 mice at 7 months post-injection, however this did not reach significance (Figure 5A, Unpaired t-test between Hu21 and Hu128/21 saline treated *P* = 0.052). Moreover, striatal volume in Hu128/21 mice tended to increase with dose of AAV5-miHTT although this was not statistically significant (Figure 5A, Supplementary Figure S8A, one-way ANOVA on Hu128/21 treated animals *P* = 0.465). However, using multiple linear regression analysis, when an interactive item between dose and genotype in a model including both Hu21 and Hu128/21 treated groups was added, we observed a significant reduction of striatal volume in saline treated Hu128/21 compared to Hu21 mice (Supplementary Figure S8B, coefficient: −0.90, *P* = 0.005). We also observed a significant dose-dependent striatal volume increase in Hu128/21 mice with AAV5-miHTT treatment (Supplementary Figure S8B, coefficient: 0.37, *P* = 0.029).

Consistent with a previous report (61), there was no significant difference in cortical volume at 9 months of age in Hu128/21 compared to Hu21 mice (Figure 5B, Unpaired t-test between Hu21 and Hu128/21 saline treated *P* = 0.6380). Moreover, there was no effect of AAV5-miHTT treatment on cortical volume (one-way ANOVA on Hu128/21 treated animals *P* = 0.9741).

Significant corpus callosum atrophy has been reported in Hu128/21 compared to Hu21 control mice as early as 6 months of age. In this study, there was a strong trend towards a reduced corpus callosum volume in Hu128/21 compared to Hu21 saline treated animals at 7 months of age but this did not reach statistical significance (Figure 5C, unpaired *t*-test between Hu21 and Hu128/21 saline treated *P* = 0.0623). Moreover, no significant effect of AAV5-miHTT treatment was observed on corpus callosum volume, however, there was a trend towards an exacerbation of corpus callosum atrophy as opposed to an amelioration with medium and high dose treatment (Figure 5C, Supplementary Figure S9A, one-way ANOVA on Hu128/21 treated animals *P* = 0.3810). Since we observed a decrease in corpus callosum volume with medium and high dose but not with low dose AAV5-miHTT treatment, we introduced a quadratic item to investigate the relationship between corpus callosum volume, dose and genotype. Using this quadratic model, we found that there was a significant effect of genotype (Supplementary Figure S9B, coefficient: −0.219, *P* = 0.017) but not dose of AAV5-miHTT on corpus callosum volume in Hu128/21 mice (Supplementary Figure S9B, coefficient: −0.033, *P* = 0.218).

Next, the effect of AAV5-miHTT treatment on DARPP-32 immunoreactivity was evaluated at 2 and 7 months post-injection. DARPP-32 is specifically expressed in medium spiny neurons of the striatum (68). Notably, we observed significantly reduced DARPP-32 integrated optical density (IOD) in Hu128/21 compared Hu21 saline animals at 2 months post-injection which was not reported previously (Figure 5D, Unpaired *t*-test between Hu21 and Hu128/21 saline treated *P* = 0.0316). This effect was rescued following treatment with AAV5-miHTT treatment at all doses (one-way ANOVA on Hu128/21 treated animals *P* = 0.0002. Tukey multiple comparison test ***P < 0.001 Hu128/21 saline versus high, medium and low doses). At 7 months post-injection, there was no significant genotypic difference in DARPP-32 IOD between Hu21 and Hu128/21 saline treated mice (unpaired *t*-test between Hu21 and Hu128/21 saline treated *P* = 0.5222). Furthermore, there was no significant effect of AAV5-miHTT treatment on Hu128/21 mice at this timepoint (one-way ANOVA on Hu128/21 treated animals *P* = 0.7315).

### AAV5-miHTT shows efficient transduction and potent suppression of wtHTT in Hu21 control mice

**Figure 6.**
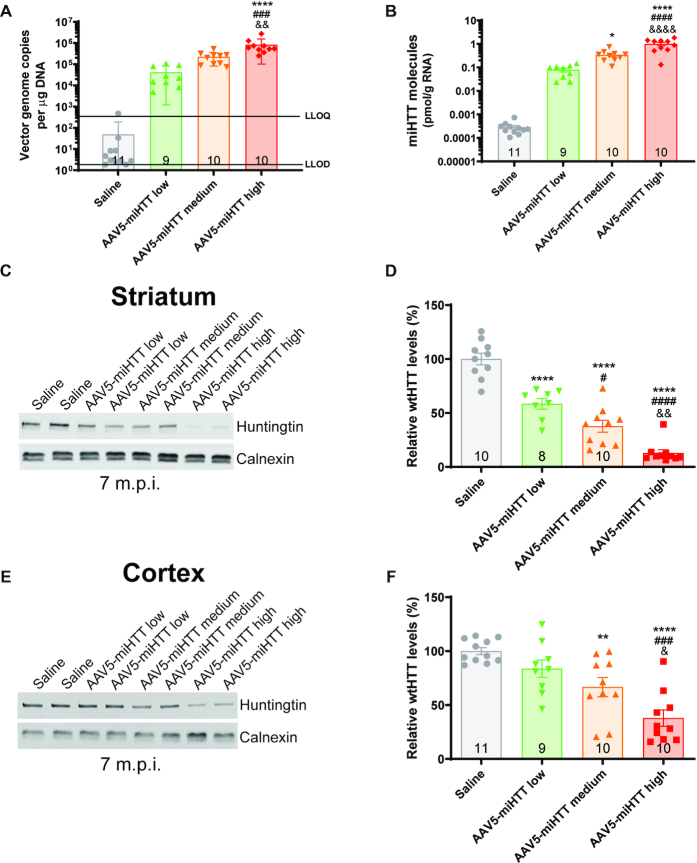
AAV5-miHTT shows efficient transduction and potent suppression of wtHTT in Hu21 control mice. (**A**) AAV5-miHTT viral vector genome copies in the cortex of Hu21 at 7 months post-injection (One way ANOVA *P*<0.0001. Tukey multiple comparison test ***P< 0.0001 high dose compared to saline, ^###^*P* = 0.0003 high compared to low dose, ^&&^*P* = 0.0044 high compared to medium dose. *N* = 9–11). LLOQ: lower limit of quantification, LLOD: lower limit of detection. (**B**) Total picomoles of mature miHTT miRNA per gram of total RNA in the cortex of Hu21 mice at 7 months post-injection (one way ANOVA *P*< 0.0001. Tukey multiple comparison test *P = 0.0387 medium and ****P< 0.0001 high dose compared to saline, ^####^*P*< 0.0001 high compared to low dose, ^&&&&^*P*< 0.0001 high compared to medium dose. *N* = 9–11). Representative western blot showing dose-dependent HTT lowering in (**C**) striatum and (**E**) cortex at 7 months post-injection. Quantification of wtHTT levels in the (**D**) striatum (one-way ANOVA *P*< 0.0001. Tukey's multiple comparison test ****P< 0.0001 saline compared to low, medium and high dose, ^#^*P* = 0.03 medium and ^####^*P*< 0.0001 high compared to low dose, ^&&^*P* = 0.0040 high compared to medium dose. *N* = 8–10) and (**F**) cortex (one-way ANOVA *P*< 0.0001. Tukey's multiple comparison test saline compared **P = 0.0082 medium and ****P< 0.0001 high dose, ^###^*P* = 0.0004 high compared to low dose, ^&^*P* = 0.0326 high compared to medium dose. *N* = 9–11) at 7 months post-injection.

To evaluate transduction efficiency of AAV5-miHTT in Hu21 mice, concentrations of viral vector DNA and mature miHTT miRNA molecules were measured in the cortex at 7 months post-injection. Consistent with results from Hu128/21 mice, we observed a significant dose-dependent increase in the number of AAV5-miHTT viral vector genome copies (Figure 6A, one-way ANOVA *P* < 0.0001. Linear regression of dose versus genome copies in Hu21 treated groups *r*^2^ = 0.3824, non-linear slope *P* < 0.0001) and mature miHTT miRNA molecules (Figure 6B, one way ANOVA *P* < 0.0001. Linear regression of dose versus pmol miHTT/g RNA in Hu21 treated groups *r*^2^ = 0.5942, non-linear slope *P* < 0.0001) at 7 months post-injection.

To evaluate target engagement and the pharmacodynamic properties of AAV5-miHTT in Hu21 control mice, levels of soluble full-length, human wtHTT were quantified in both the striatum and cortex at 7 months post-injection. In the striatum, we observed a dose-dependent reduction of wtHTT with low, medium and high dose treatments resulting in 42%, 62% and 87% suppression at 7 months post-injection, respectively (Figure 6C and D, one-way ANOVA *P* < 0.0001. Linear regression of dose vs HTT levels in Hu21 treated groups *r*^2^ = 0.7170, non-linear slope *P* < 0.0001). Moreover, a dose-dependent reduction of wtHTT was also observed in the cortex with low, medium and high dose treatments resulting in 16%, 33% and 62% reduction at 7 months post-injection, respectively (Figure 6E and F, one-way ANOVA *P* < 0.0001. Linear regression of dose versus HTT levels in Hu21 treated groups *r*^2^ = 0.5341, non-linear slope *P* < 0.0001).

Consistent with intrastriatal delivery of AAV5-miHTT (and results from the Hu128/21 mice), we observed a significantly higher magnitude of HTT suppression in the striatum compared to the cortex at 7 months post-injection (Two-way ANOVA brain region *P* < 0.0001, dose *P* < 0.0001, interaction *P* = 0.0673. Bonferroni post-test *P < 0.05 low dose, **P < 0.01 medium dose and *P < 0.05 high dose in striatum compared to cortex).

### AAV5-miHTT treatment may induce motor but not psychiatric or cognitive abnormalities in Hu21 control mice

**Figure 7.**
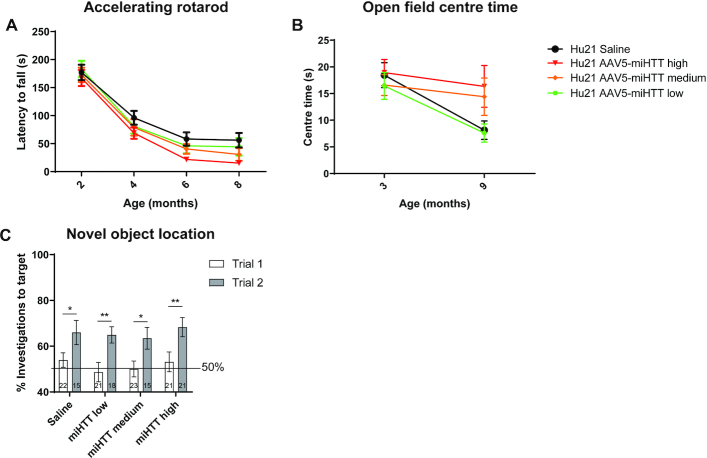
AAV5-miHTT treatment may induce motor but not psychiatric or cognitive abnormalities in Hu21 control mice. (**A**) Accelerating rotarod test of Hu21 treated groups at 2, 4, 6 and 8 months of age (mixed-effects model on all Hu21 groups treatment *P* = 0.1719, timepoint *P*< 0.0001, treatment × timepoint *P* = 0.7945. 2 months: *N* = 24–26, 4 months: *N* = 24–26, 6 months: *N* = 24–25, 8 months: *N* = 23–25). (**B**) Open field centre time at 1 and 7 months post-injection (mixed effects model on all Hu21 groups treatment *P* = 0.2531, timepoint *P*< 0.0001, treatment × timepoint *P* = 0.1642. 3 months: *N* = 24–26, 9 months: *N* = 17–25). (**C**) Novel object location test at 9 months of age comparing Hu21 treated groups (unpaired *t*-test of trial 1 versus trial 2 for saline *P = 0.0227, low dose **P = 0.0034, medium dose *P = 0.0126, high dose **P = 0.0081. Trial 1, *N* = 21–23; trial 2, *N* = 15–21).

To evaluate the effect of long-term suppression of wtHTT on behaviour in Hu21 mice we looked at motor, psychiatric and cognitive phenotypes following treatment with AAV5-miHTT. We observed a significant impairment of motor coordination in Hu21 mice over time and there was a trend towards reduction in the latency to fall from the accelerating rotarod as a result of AAV5-miHTT treatment (Figure 7A, mixed-effects model on all Hu21 groups at all timepoints; treatment *P* = 0.1719, timepoint *P* < 0.0001, treatment × timepoint *P* = 0.7945). Moreover, there was a strong trend towards AAV5-miHTT treatment-induced impairment of motor coordination at 4 (one-way ANOVA *P* = 0.0895) and 6 months post-injection (one-way ANOVA *P* = 0.0834) in Hu21 mice.

No significant effect of AAV5-miHTT treatment was observed on anxiety-like phenotypes in Hu21 mice as measured by time spent in the centre of the open field but there was a significant effect associated with age of animals at time of test (Figure 7B, mixed effects model on all Hu21 groups time *P* < 0.0001, treatment *P* = 0.2531, time × treatment *P* = 0.1642). There was a strong trend towards maintaining a high centre time from 3 to 9 months of age following treatment with medium and high dose of AAV5-miHTT. However, due to the abnormal anxiety-like behavior of the saline treated Hu21 group, this finding is difficult to interpret.

Lastly, we did not observe an effect of AAV5-miHTT treatment on preference for a known object in a novel location at 7 months post-injection (Figure 7C, Unpaired t-test on Hu21 AAV5-miHTT treated groups *P* = 0.0081, *P* = 0.0126, and *P* = 0.0034 for high, medium, and low dose treated groups, respectively).

### AAV5-miHTT treatment may induce striatal atrophy in Hu21 control mice

**Figure 8.**
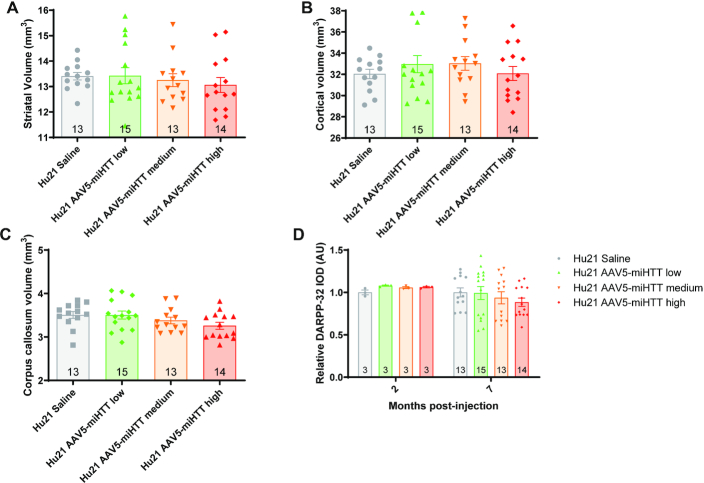
AAV5-miHTT treatment may induce striatal atrophy in Hu21 control mice. Stereological evaluation of (**A**) striatal (one-way ANOVA *P* = 0.533. *N* = 13–15), (**B**) cortical (one-way ANOVA *P* = 0.5855. *N* = 13–15) and (**C**) corpus callosum (one-way ANOVA *P* = 0.1176. *N* = 13–15) volumes was performed at 7 months post-injection. (**D**) IOD of DARPP-32 immunoreactivity was evaluated in a series of 4 mid-striatal sections at 2 months post-injection (one-way ANOVA *P* = 0.0351. *N* = 3) and 7 months post-injection (one-way ANOVA *P* = 0.5430. *N* = 13–15). Values were normalized to the mean value for saline treated Hu21 animals at each timepoint.

To further evaluate the tolerability of long-term wtHTT suppression in the wild type context, we next looked at the effect of AAV5-miHTT on region-specific volumetric changes and DARPP-32 immunoreactivity in the Hu21 mice. We did not observe a significant effect of AAV5-miHTT treatment on striatal volume at 7 months post-injection (Figure 8A, Supplementary Figure S8A, one-way ANOVA on Hu21 treated groups *P* = 0.533). However, multiple linear regression analysis revealed that there was a significant dose-dependent decrease in striatal volume with AAV5-miHTT treatment (Supplementary Figure S8B, coefficient: −0.55, *P* = 0.039).

We did not observe a significant effect of AAV5-miHTT treatment on cortical volume in Hu21 mice, however there was a modest trend towards increased volume at all doses of AAV5-miHTT (Figure 8B, one-way ANOVA *P* = 0.5855). Lastly, there was no significant effect of treatment on corpus callosum volume in Hu21 mice, however, there was a trend towards volume reduction with medium and high doses of AAV5-miHTT (Figure 8C, Supplementary Figure S9A, one-way ANOVA *P* = 0.1176). Moreover, quadratic modeling also confirmed that there was no significant effect of AAV5-miHTT dose on corpus callosum volume in Hu21 mice (Supplementary Figure S9B, coefficient: −0.031, *P* = 0.78).

Additionally, we evaluated the effect of AAV5-miHTT on DARPP-32 IOD in the Hu21 mice at 2 and 7 months post-injection. There was a significant effect of AAV5-miHTT treatment on DARPP-32 IOD at 2 months post-injection (Figure 8D, one-way ANOVA *P* = 0.0351). In contrast, at 7 months post-injection there was no significant effect of AAV5-miHTT treatment but there was a trend towards reduced DARPP-32 IOD at the medium and high doses (Figure 8D, one-way ANOVA *P* = 0.5430).

## DISCUSSION

We have previously demonstrated tolerability of AAV5-miHTT and potent mHTT suppression in an acute rat model of HD (62) and in a large model of HD, the transgenic HD minipig (63), as well as functional improvement of HD-like phenotypes in R6/2 mice (64). Here, we aimed to examine the tolerability and therapeutic efficacy of long-term non-selective HTT lowering following direct intrastriatal administration of increasing doses of AAV5-miHTT in the humanized Hu128/21 mouse model of HD. Additionally, we sought to examine the effects of long term wtHTT suppression with AAV5-miHTT in the humanized Hu21 control mouse model.

In this study, intrastriatal infusions of AAV5-GFP by CED resulted in robust transduction at the infusion site as well as in distal brain regions. We observed broad distribution of AAV5 throughout the basal ganglia, deeper layers of the cortex and hippocampus. These findings are consistent with reports that AAV5 undergoes anterograde and retrograde axonal transport following intrastriatal infusion (66) resulting in broad transduction to many of the structures affected in HD in a large animal model of HD (63). Immunohistochemical analysis at the infusion site of AAV5-miScr injected animals revealed that medium spiny neurons of the striatum were efficiently transduced (GFP+/DARPP-32+: 47.22±6.71%). Moreover, we measured transduction of neurons (GFP+/NeuN+: 53.06 ± 7.14%), astrocytes/neural progenitors (GFP+/GFAP+: 37.20 ± 7.62%) and to a lesser extent of microglia (GFP+/Iba1+: 3.88 ± 0.97%) at the infusion site. It is important to note that co-localization was assessed using immunohistochemistry and confocal imaging at a relatively low magnification to capture many cells. As a result, cells transduced with smaller numbers of viral particles may express the GFP transgene at levels below the limit of quantification, too dim to be identified at the magnification used, or below the background fluorescence level. Considering the potency of HTT suppression observed, and the limitations of immunohistochemistry for quantitative assays, we believe that the transduction efficiency may be substantially higher than was measured. Additionally, we qualitatively observed transduction of NeuN, GFAP and Iba1 positive cells at regions more distal from the site of infusion, such as the cortex and the hippocampus. These findings are important as increasing evidence implicates glial cells as important contributors to HD pathogenesis (69–72).

Tolerability in this study was evaluated using complementary measures of gliosis, brain morphology, body weight and survival. We observed that high dose AAV5-miHTT induced astrogliosis in both Hu21 and Hu128/21 mice, which may indicate potential toxicity of this dose in the humanized mice. However, astrogliosis was not observed following medium or low dose AAV5-miHTT treatment and even high dose AAV5-miHTT treatment did not induce microgliosis, abnormal striatal morphology or lateral ventricle volume increases at 2 months post-injection at any dose. Moreover, there was no significant effect of AAV5-miHTT treatment on body weight or survival in either the Hu128/21 or Hu21 mice up to 7 months post-injection at any of the doses tested. In this context, astrogliosis without accompanying microgliosis and reduction in body weight and survival in both genotypes suggests that treatment was generally well tolerated for the duration of this study. Notably, we did not investigate glial activation at later timepoints post-injection to determine if the high dose effect of AAV5-miHTT was transient or if it was sustained for the duration of the study.

The Hu128/21 model represents a genetically accurate model of HD, expressing both a mutant and a wild type full-length human *HTT* transgene on a mouse *Htt* nullzygous background. Thus, it is an appropriate model to evaluate target engagement with a human sequence-specific targeting therapeutic, such as AAV5-miHTT. We observed a dose-dependent suppression of HTT with AAV5-miHTT in both the striatum (the site of injection) and the cortex, that was sustained up to 7 months post-injection.

We observed a trend towards time-dependent effect of treatment on levels of HTT in the striatum of Hu128/21 mice with AAV5-miHTT treatment reaching the highest magnitude of HTT suppression at 7 months post-injection. This suggests that HTT lowering may extend well beyond 7 months post-injection and that further reduction of HTT may occur with extended treatment. Previous reports have shown that AAVs similar to the one used in this study can induce long term expression of a transgene for multiple years in non-human primates (73,74) suggesting that HTT suppression with AAV5-miHTT could be sustained long term following a single administration. A single treatment with AAV5-miHTT results in sustained HTT lowering, indicating that a more invasive method of delivery may be acceptable. In the clinical setting, direct intracranial CED administration of AAV5-miHTT into the striatum will be performed, which has previously been shown in a large animal model of HD to efficiently lower HTT in the areas most affected by the disease (63). These results are encouraging in the context of a therapy for HD where suppression of mHTT would likely need to be achieved in the striatum and cortex and maintained throughout the lifetime of an individual.

In this study, we also investigated the effect of long term wtHTT lowering in Hu21 control mice. We observed a significant dose-dependent reduction of wtHTT in both the striatum and cortex with AAV5-miHTT treatment up to 7 months post-injection. Notably, AAV5-miHTT treatment had no effect on body weight or survival up to 7 months post-injection. Inactivation of the mouse *Htt* homolog at 3 months of age has previously been shown to decrease survival from 12 months of age (9 months post-inactivation) (51) so it remains a possibility that the treatment paradigm in this study was not sufficiently long to detect potential detrimental effects of wtHTT suppression on survival.

Potent, non-selective HTT lowering with AAV5-miHTT improved selected modest behavioural phenotypes in Hu128/21 mice. We found that high dose AAV5-miHTT treatment resulted in increased time in the centre of an open field suggesting a potential anxiolytic effect of treatment. This is consistent with the anxiolytic effect following late treatment with a non-selective human specific ASO in the Hu97/18 humanized model (28). However, in the present study, the saline treated Hu21 mice exhibited abnormal anxiety-like behavior, which confounds interpretation of this data. Furthermore, we observed that Hu128/21 mice showed a spatial learning deficit compared to Hu21 mice, showing no preference to investigate a known object in a novel location. Treatment with AAV5-miHTT medium and high dose prevented development of this deficit, resulting in restored preference for the target object. These data suggest benefit of treatment on spatial learning deficits in Hu128/21 mice. These findings are also consistent with the observed effects of ASO-mediated non-selective HTT lowering in Hu97/18 mice (28).

Consistent with a previous study, we observed a significant impairment of motor coordination in both genotypes over time with Hu128/21 mice performing significantly worse on the accelerating rotarod compared to Hu21 control animals (61). This progressive motor deficit has been attributed to a dramatic body weight increase in both genotypes, suggesting that this model is not well suited for evaluating the therapeutic efficacy of AAV5-miHTT using motor readouts. We did not observe any effects of AAV5-miHTT treatment on motor performance in the Hu128/21 mice. This is similar to ASO-mediated HTT suppression in Hu97/18 mice, in which progressive weight gain-induced motor deficits in the control genotype confounded assessment of the effects of treatment on longitudinal accelerating rotarod performance (28). In contrast, AAV5-miHTT treatment was shown to improve motor coordination in the R6/2 mouse model as measured by latency to fall from the rotarod (64). The R6/2 model shows the characteristic weight loss phenotype associated with HD (75). In the Hu21 control mice, we observed a trend towards reduction in the latency to fall from the accelerating rotarod with AAV5-miHTT treatment. This result must be cautiously interpreted given the progressive decline of saline treated Hu21 mice over time but may suggest a potential detrimental effect of long-term suppression of wtHTT on motor coordination. This is consistent with longitudinal rotarod performance deficits observed following ablation of *Htt* from 3 months of age (51).

Hu128/21 mice develop striatal atrophy as early as 3 months of age (61). Using multiple linear regression analysis, we observed a significant reduction of striatal volume in saline treated Hu128/21 compared to Hu21 mice. While the effects of AAV5-miHTT treatment on Hu128/21 striatal volume failed to reach significance at any dose by ANOVA, there was a significant dose-dependent striatal volume increase with AAV5-miHTT treatment in Hu128/21 mice at 9 months of age. These results suggest that AAV5-miHTT treatment may reduce striatal atrophy in an HD mouse model. In contrast, we observed that AAV5-miHTT treatment led to a significant dose-dependent striatal volume reduction in Hu21 control mice, though again this effect was not significant at any dose considered independently. This finding is consistent with brain atrophy caused by long term *Htt* elimination in the adult mouse (51). Our findings suggest a potential detrimental effect of long-term loss of wtHTT in a non-HD context. However, our data also suggests that in the context of HD, the therapeutic benefits of mHTT reduction may outweigh the potentially detrimental effects of wtHTT loss following non-selective HTT lowering.

Corpus callosum atrophy is also a feature of Hu128/21 mice which is apparent from 6 months of age (61). Multiple regression analysis demonstrated the expected atrophy in Hu128/21 mice at 9 months of age. However, we observed no effect of AAV5-miHTT treatment in either Hu128/21 or Hu21 mice. This is perhaps not surprising, as we did not observe transgene expression in this white matter structure. We also observed reduced striatal DARPP-32 at 2 months post-injection in Hu128/21 brains and a trend toward reduction at 7 months post-injection. This is consistent with a previous report showing a persistent trend towards reduced striatal DARPP-32 in Hu128/21 mice (61). AAV5-miHTT treatment rescued loss of DARPP-32 immunoreactivity at 2 months post-injection. Interestingly, at 7 months post-injection low and medium dose AAV5-miHTT treatment reversed the trend towards reduced DARPP-32 observed in Hu128/21 striata, while high dose did not. Additionally, the medium and high doses induced a trend toward loss of striatal DARPP-32. Notably, a previous report showed that DARPP-32 expression was not affected by *Htt* ablation in the adult mouse (51). ASO-mediated non-selective HTT suppression has been shown to prevent striatal DARPP-32 loss in Hu97/18 brain if assessed during the period of HTT suppression, but not once HTT expression is restored (28). In contrast, allele-selective ASO-mediated HTT suppression was shown to restore striatal DARPP-32 both during HTT suppression and long after restoration of HTT expression. Taken together, these data suggest a complex relationship between HTT lowering and striatal DARPP-32, and may again suggest that non-selective HTT suppression can show benefit in HD animals and modest detriment in control animals, and perhaps indicating that the presence of mutant HTT may be more toxic than the potential effects associated with loss of wtHTT. We did not look at thalamic calcification or brain iron homeostasis in the AAV5-miHTT treated Hu21 mice which has been reported in animals following long term *Htt* elimination (51).

With non-selective HTT lowering approaches in clinical development, many questions surrounding how much wtHTT lowering will be tolerated long-term and how much mHTT suppression (and in what brain regions) is required to show clinical efficacy remain to be answered. In Hu128/21 mice, we found that low dose AAV5-miHTT, which resulted in ∼60% lowering of mHTT and ∼67% lowering of wtHTT in the striatum as well as ∼30% lowering of mHTT and ∼40% lowering of wtHTT in cortex at 7 months post-injection had an effect on preventing striatal atrophy but not on behavioural phenotypes. With medium dose AAV5-miHTT, which resulted in ∼75% lowering of mHTT and ∼80% lowering of wtHTT in the striatum as well as ∼40% lowering of mHTT and ∼50% lowering of wtHTT in the cortex at 7 months post-injection, this level of HTT suppression led to improvement of striatal atrophy but also resulted in a trend towards preventing the onset of spatial learning deficits in Hu128/21 mice. Lastly, with high dose AAV5-miHTT treatment, which resulted in ∼90% lowering of mHTT and ∼95% lowering of wtHTT in the striatum as well as ∼65% lowering of mHTT and ∼ wtHTT in the cortex at 7 months post-injection, this magnitude of HTT suppression improved striatal atrophy, prevented onset of spatial learning deficits and blunted an anxiety-like phenotype in Hu128/21 mice. However, despite these beneficial effects observed with high dose AAV5-miHTT, this dose also resulted in astrogliosis. These data suggest that a certain threshold of mHTT lowering may be required to have an impact on behavioural phenotypes via non-selective HTT lowering. However, these findings need to be interpreted cautiously. In this study, we achieved more uniform distribution of AAV5-miHTT in the striatum compared to the cortex, with deeper layers of the cortex receiving more viral particles than more superficial layers. Therefore, our interpretation of the magnitude of HTT lowering in a cortical homogenate represents an average of HTT levels, with some cells receiving many copies of AAV5-miHTT and others receiving very few. This makes it challenging to correlate the magnitude of HTT lowering in the cortex required to induce benefits in behavioural and neuropathological phenotypes.

Longer duration preclinical studies in large animal models of HD are required to evaluate the functional consequences of high magnitude HTT suppression in the adult striatum as well as the threshold of wtHTT lowering that is tolerated. The first-in-human phase 1/2a clinical trial evaluating the non-selective HTT lowering ASO, HTT_RX_ (RG6042, RO7234292), demonstrated a dose-dependent reduction of mHTT in the CSF of treated individuals (53). We have previously demonstrated that the reduction of mHTT levels in the brain of HD mice following intracerebroventricular injection of an ASO was reflected by a correlative reduction of mHTT in the CSF, suggesting that levels of mHTT in CSF could be used as a pharmacodynamic biomarker to assess target engagement in the CNS (76). However, the correlation of mHTT levels in the brain and CSF in humans remains unknown. Notably, a mean mHTT reduction of up to 42% in the CSF of patients (maximum individual reduction of 63%) was found to be safe and well tolerated for up to 4 months (113 days) (53). With an open label extension (NCT03342053) and an efficacy trial (NCT03761849) underway, these clinical data will help to bring new insights into the tolerability of long-term non-selective HTT lowering in humans.

In this study, we demonstrate that intrastriatal delivery of AAV5-miHTT results in potent suppression of HTT in both the striatum and cortex of Hu128/21 mice that is sustained for at least 7 months post-injection. Importantly, we demonstrate that this magnitude of non-selective suppression of HTT in Hu128/21 mice leads to improvements of select behavioural and neuropathological HD-like phenotypes. We also demonstrate that potent reduction of wtHTT with AAV5-miHTT in Hu21 control mice does not induce psychiatric or cognitive behavioral deficits. We did however observe potential detrimental effects of wtHTT lowering in Hu21 mice on motor coordination and striatal atrophy. Taken together, these data suggest that the benefits of mHTT reduction following non-selective HTT suppression may outweigh the potential negative effects related to loss of wtHTT. In conjunction with additional tolerability studies in large animal models, this data supports the translation of AAV5-miHTT from preclinical to clinical trials for the treatment of HD.

## Supplementary Material

gkz976_Supplemental_File
