# Supplementary material for: Potent and sustained huntingtin lowering via AAV5 encoding miRNA preserves striatal volume and cognitive function in a humanized mouse model of Huntington disease
Source: Nucleic Acids Res. 2019 Nov 20;48(1):36–54. doi: 10.1093/nar/gkz976 (PMC7145682; doi:10.1093/nar/gkz976)
Supplement: gkz976_Supplemental_File [file gkz976_supplemental_file.docx]

**Supplementary data**

**Supplementary figure S1. AAV5-GFP, AAV5-miScr and AAV5-miHTT high dose induced astrogliosis in Hu21 and Hu128/21 mice.** Representative images of the striatum and corpus callosum from similar coronal sections stained for GFAP (red) to evaluate astrocytic neuroinflammation at 4 months of age (2 months post-treatment). Consistent imaging settings were maintained between animals and time-points to allow for qualitative comparison.

**Supplementary figure S2. AAV5-GFP and AAV5-miScr induce microgliosis in Hu21 and Hu128/21 mice.** Representative images of the striatum from similar coronal sections stained for Iba1 (red) to evaluate microglial neuroinflammation at 4 months of age (2 months post-treatment). Consistent imaging settings were maintained between animals and time-points to allow for qualitative comparison.

**Supplementary figure S3. AAV5-GFP and AAV5-miScr cause abnormal DARPP-32 staining in Hu21 and Hu128/21 mice.** Representative images of mid-striatal sections stained for DARPP-32 to evaluate tolerability in mice treated with **(A)** AAV5-GFP, **(B)** miScr and **(C)** miHTT viruses at 4 months of age (2 months post-injection).

|  |  |
| --- | --- |

**Supplementary figure S4. AAV5-GFP and AAV5-miScr treatment causes increased lateral ventricle size.** Mean lateral ventricle area was evaluated in a series of mid-striatal sections as a measure of gross CNS tolerability at 2 months post-injection. Area was normalized to the mean value for saline treated animals of the same genotype (Hu21 treated groups: One-way ANOVA p<0.0001. Tukey's multiple comparison post hoc analysis **p=0.0040, ****p<0.0001; Hu128/21 treated groups: One-way ANOVA p=0.0036. Tukey's multiple comparison post hoc analysis **p=0.0017).

**Supplementary figure S5. AAV5-miHTT treatment does not affect body weight or survival up to 7 months post-injection.** Longitudinal body weight of **(A)** Hu21 (Mixed-effects model treatment p=0.4077, age p<0.0001, treatment x age p=0.6130) and **(B)** Hu128/21 (Mixed-effects model treatment p=0.0997, age p<0.0001, treatment x age p=0.1509) treated mice. Survival proportions of treated **(C)** Hu21 (Log-rank test: Chi square=0.0005084, df=3, p>0.9999) and **(D)** Hu128/21 (Log-rank test: Chi square=3.056, df=3, p=0.3831) until time of collection at 9 months of age (7 months post-injection).

**Supplementary figure S6:** Western blots used for quantification of HTT suppression with AAV5-miHTT in Hu21 and Hu128/21 mice. HTT lowering in Hu128/21 at **(A)** 1 month post-injection, **(B)** 4 months post-injection, **(C)** 7 months post-injection and **(D)** Hu21 7 month post-injection.

**Supplementary figure S7:** **AAV5-miHTT treatment does not improve motor phenotypes in Hu128/21 mice.** Accelerating rotarod test of Hu21 saline vs Hu128/21 treated groups at 2, 4, 6 and 8 months of age shows a significant decrease in latency to fall between genotypes (Mixed-effects model of Hu21 and Hu128/21 saline groups at all timepoints: genotype p=0.0161, timepoint p<0.0001, genotype x timepoint p=0.1839) but no effect of treatment (Mixed-effects model of all Hu128/21 treated groups at all timepoints: treatment p=0.5549, timepoint p<0.0001, treatment x timepoint p= 0.8614. 2 months: N=26-33, 4 months: N=26-33, 6 months: N=25-32, 8 months: N=21-25).

**Supplementary figure S8: AAV5-miHTT treatment shows dose-dependent effects on striatal volume in Hu21 and Hu128/21 mice.** (A) Muliple linear regression comparing the striatal volume of Hu21 and Hu128/21 in relation to dose of viral genomes (dots indicate mean values and spikes reflect SEs). **(B)** Modeled relationship between striatal volume and dose and genotype (Saline Hu21 vs Hu128/21: coefficient -0.90, p=0.005; Hu21 vs AAV5-miHTT dose: coefficient -0.55, p=0.039; Hu128/21 vs AAV5-miHTT dose: coefficient 0.37, p=0.029).

**Supplementary figure S9: AAV5-miHTT treatment does not significantly alter corpus callosum volume in either Hu128/21 or Hu21 mice. (A)** Comparison of corpus callosum volume between Hu21 and Hu128/21 in relation to dose of viral genomes (dots indicate mean values and spikes reflect SEs). **(B)** Modeled relationship between corpus callosum volume and dose and genotype (Saline Hu21 vs Hu128/21: coefficient -0.219, p=0.017; Hu21 vs AAV5-miHTT dose: coefficient -0.031, p=0.78; Hu128/21 vs AAV5-miHTT dose: coefficient -0.033, p=0.218).

**Supplementary table S1:** One-way ANOVAs with Tukey’s multiple-comparison corrections for each of the behavioural, neuropathological and biochemical tests performed in this study. P value for each genotype compared to saline treated of the same genotype. Significant p-values are bolded.

| **Survival** | **Hu21 AAV5-miHTT vs Saline** | | | **Hu128/21 AAV5-miHTT vs Saline** | | |  |  |  |  |
| --- | --- | --- | --- | --- | --- | --- | --- | --- | --- | --- |
| **Treatment** | **Chi Square** | **DF** | **p value** | **Chi Square** | **DF** | **p value** |  |  |  |  |
| AAV5-miHTT low | 0.0001923 | 1 | 0.9889 | 0.6154 | 1 | 0.4328 |  |  |  |  |
| AAV5-miHTT medium | 0.0001923 | 1 | 0.9889 | 0.5665 | 1 | 0.4516 |  |  |  |  |
| AAV5-miHTT high | 0.0001923 | 1 | 0.9889 | 3.104 | 1 | 0.0781 |  |  |  |  |
| **Vector genome copies** | **Hu21** | | | | | **Hu128/21** | | | | |
| **Treatment** | **Age** | **Mean** | **SD** | **N** | **p value** | **Age** | **Mean** | **SD** | **N** | **p value** |
| Saline | 9m | 49.10181818 | 139.9050811 | 11 |  | 9m | 51.5 | 94.06700803 | 7 |  |
| AAV5-miHTT low | 9m | 41681.11111 | 40464.09286 | 9 | 0.9944 | 9m | 105855.5556 | 98609.81329 | 9 | 0.9997 |
| AAV5-miHTT medium | 9m | 223280 | 140588.6656 | 10 | 0.5178 | 9m | 1583750 | 878817.184 | 8 | 0.5743 |
| AAV5-miHTT high | 9m | 826300 | 724262.0842 | 10 | **<0.0001** | 9m | 4775000 | 4427215.547 | 9 | **0.0021** |
| **Mature miRNA pmol/g** | **Hu21** | | | | | **Hu128/21** | | | | |
| **Treatment** | **Age** | **Mean** | **SD** | **N** | **p value** | **Age** | **Mean** | **SD** | **N** | **p value** |
| Saline | 9m | 0.000284 | 0.000173209 | 11 |  | 9m | 0.000451375 | 0.000314305 | 8 |  |
| AAV5-miHTT low | 9m | 0.079144444 | 0.04458966 | 9 | 0.922 | 9m | 0.193666667 | 0.058927922 | 9 | 0.2434 |
| AAV5-miHTT medium | 9m | 0.3418 | 0.174340535 | 10 | **0.0387** | 9m | 0.61925 | 0.36672479 | 8 | **<0.0001** |
| AAV5-miHTT high | 9m | 0.9993 | 0.528058719 | 10 | **<0.0001** | 9m | 1.003375 | 0.199618878 | 8 | **<0.0001** |
| **Hu128/21 HTT levels - Striatum** | **wtHTT** | | | | | **mHTT** | | | | |
| **Treatment** | **Age** | **Mean** | **SD** | **N** | **p value** | **Age** | **Mean** | **SD** | **N** | **p value** |
| Saline | 3m | 100 | 8.61 | 3 |  | 3m | 100 | 13.3 | 3 |  |
| AAV5-miHTT low | 3m | 56.82 | 26.18305368 | 3 | 0.0539 | 3m | 67.45 | 39.57904496 | 3 | 0.4319 |
| AAV5-miHTT medium | 3m | 42.35333333 | 8.289036936 | 3 | **0.0127** | 3m | 46.78 | 14.6685923 | 3 | 0.1153 |
| AAV5-miHTT high | 3m | 27.66 | 17.30173113 | 3 | **0.0033** | 3m | 30.51 | 23.17304253 | 3 | **0.0375** |
| **Treatment** | **Age** | **Mean** | **SD** | **N** | **p value** | **Age** | **Mean** | **SD** | **N** | **p value** |
| Saline | 6m | 100 | 57.08220633 | 6 |  | 6m | 100 | 57.66120568 | 6 |  |
| AAV5-miHTT low | 6m | 63.1775 | 34.98768581 | 4 | 0.4923 | 6m | 49.0625 | 17.98520943 | 4 | 0.1911 |
| AAV5-miHTT medium | 6m | 20.8 | 4.114608122 | 3 | 0.0583 | 6m | 15.05 | 1.343465668 | 3 | **0.0278** |
| AAV5-miHTT high | 6m | 9.7775 | 5.872769789 | 4 | **0.0163** | 6m | 7.7775 | 3.741945795 | 4 | **0.009** |
| **Treatment** | **Age** | **Mean** | **SD** | **N** | **p value** | **Age** | **Mean** | **SD** | **N** | **p value** |
| Saline | 9m | 100 | 17.16314423 | 6 |  | 9m | 100 | 22.76319105 | 7 |  |
| AAV5-miHTT low | 9m | 32.33428571 | 13.04105678 | 7 | **<0.0001** | 9m | 41.25142857 | 14.44699211 | 7 | **<0.0001** |
| AAV5-miHTT medium | 9m | 18.1225 | 10.28818844 | 8 | **<0.0001** | 9m | 24.2025 | 12.21463145 | 8 | **<0.0001** |
| AAV5-miHTT high | 9m | 5.737777778 | 1.578114205 | 9 | **<0.0001** | 9m | 9.171111111 | 3.592340617 | 9 | **<0.0001** |
| **Hu128/21 HTT levels - Cortex** | **wtHTT** | | | | | **mHTT** | | | | |
| **Treatment** | **Age** | **Mean** | **SD** | **N** | **p value** | **Age** | **Mean** | **SD** | **N** | **p value** |
| Saline | 3m | 100 | 2.3 | 3 |  | 3m | 100 | 1.44 | 3 |  |
| AAV5-miHTT low | 3m | 73.75666667 | 16.69237051 | 3 | 0.2323 | 3m | 76.79 | 18.91778793 | 3 | 0.355 |
| AAV5-miHTT medium | 3m | 62.45 | 18.56808822 | 3 | 0.0664 | 3m | 65.22 | 21.0854713 | 3 | 0.1101 |
| AAV5-miHTT high | 3m | 49.90333333 | 17.61346171 | 3 | **0.0165** | 3m | 55.67333333 | 15.31201271 | 3 | **0.0396** |
| **Treatment** | **Age** | **Mean** | **SD** | **N** | **p value** | **Age** | **Mean** | **SD** | **N** | **p value** |
| Saline | 6m | 100 | 12.08273713 | 6 |  | 6m | 100 | 19.03197038 | 6 |  |
| AAV5-miHTT low | 6m | 97.615 | 16.17593583 | 4 | 0.9945 | 6m | 99.7225 | 17.28504821 | 4 | >0.9999 |
| AAV5-miHTT medium | 6m | 79.55 | 23.52609827 | 3 | 0.2627 | 6m | 67.05333333 | 14.76562336 | 3 | 0.0535 |
| AAV5-miHTT high | 6m | 44.7475 | 9.871438176 | 4 | **0.0004** | 6m | 38.2325 | 8.571045735 | 4 | **0.0002** |
| **Treatment** | **Age** | **Mean** | **SD** | **N** | **p value** | **Age** | **Mean** | **SD** | **N** | **p value** |
| Saline | 9m | 100 | 3.416758698 | 6 |  | 9m | 100 | 11.7949622 | 7 |  |
| AAV5-miHTT low | 9m | 60.64571429 | 24.92727747 | 7 | **0.0122** | 9m | 71.17285714 | 25.06791955 | 7 | 0.0546 |
| AAV5-miHTT medium | 9m | 51.48 | 19.30242472 | 8 | **0.0013** | 9m | 61.22875 | 20.35140742 | 8 | **0.0045** |
| AAV5-miHTT high | 9m | 34.20222222 | 25.35525723 | 9 | **<0.0001** | 9m | 37.87111111 | 20.31251427 | 9 | **<0.0001** |
| **Hu21 HTT levels** | **Striatum** | | | | | **Cortex** | | | | |
| **Treatment** | **Age** | **Mean** | **SD** | **N** | **p value** | **Age** | **Mean** | **SD** | **N** | **p value** |
| Saline | 9m | 100.001 | 17.36725044 | 10 |  | 9m | 99.99909091 | 10.51187277 | 11 |  |
| AAV5-miHTT low | 9m | 58.4625 | 13.70900512 | 8 | **<0.0001** | 9m | 83.71444444 | 23.97485459 | 9 | 0.3793 |
| AAV5-miHTT medium | 9m | 37.699 | 17.59563105 | 10 | **<0.0001** | 9m | 66.634 | 28.11493008 | 10 | **0.0082** |
| AAV5-miHTT high | 9m | 12.852 | 9.579424478 | 10 | **<0.0001** | 9m | 37.914 | 23.89295721 | 10 | **<0.0001** |
| **Body weight** | **Hu21** | | | | | **Hu128/21** | | | | |
| **Treatment** | **Age** | **Mean** | **SD** | **N** | **p value** | **Age** | **Mean** | **SD** | **N** | **p value** |
| Saline | 2m | 25.27 | 3.06 | 26 |  | 2m | 27.41290323 | 3.105880652 | 31 |  |
| AAV5-miHTT low | 2m | 25.86 | 2.57 | 25 | 0.8605 | 2m | 28.20606061 | 3.566417968 | 33 | 0.7764 |
| AAV5-miHTT medium | 2m | 25.71 | 2.08 | 24 | 0.9383 | 2m | 28.209375 | 2.860674337 | 32 | 0.7781 |
| AAV5-miHTT high | 2m | 25.89 | 2.87 | 25 | 0.8457 | 2m | 26.871875 | 3.696226007 | 32 | 0.9171 |
| **Treatment** | **Age** | **Mean** | **SD** | **N** | **p value** | **Age** | **Mean** | **SD** | **N** | **p value** |
| Saline | 4m | 33.90 | 5.22 | 26 |  | 4m | 38.60645161 | 4.864629858 | 31 |  |
| AAV5-miHTT low | 4m | 34.87 | 3.20 | 25 | 0.8501 | 4m | 39.37272727 | 6.580571438 | 33 | 0.9524 |
| AAV5-miHTT medium | 4m | 35.68 | 3.88 | 24 | 0.455 | 4m | 41.234375 | 5.923033735 | 32 | 0.2811 |
| AAV5-miHTT high | 4m | 35.86 | 4.40 | 25 | 0.3588 | 4m | 38.1875 | 5.698712362 | 32 | 0.9918 |
| **Treatment** | **Age** | **Mean** | **SD** | **N** | **p value** | **Age** | **Mean** | **SD** | **N** | **p value** |
| Saline | 6m | 40.09 | 6.79 | 25 |  | 6m | 44.84333333 | 6.255958998 | 30 |  |
| AAV5-miHTT low | 6m | 41.34 | 4.72 | 25 | 0.8571 | 6m | 45.290625 | 7.274141536 | 32 | 0.993 |
| AAV5-miHTT medium | 6m | 41.62 | 5.16 | 24 | 0.7711 | 6m | 48.21724138 | 5.353041123 | 29 | 0.1948 |
| AAV5-miHTT high | 6m | 42.47 | 5.33 | 25 | 0.432 | 6m | 44.44375 | 6.790882764 | 32 | 0.995 |
| **Treatment** | **Age** | **Mean** | **SD** | **N** | **p value** | **Age** | **Mean** | **SD** | **N** | **p value** |
| Saline | 8m | 42.79 | 7.74 | 25 |  | 8m | 47.81428571 | 6.490091349 | 21 |  |
| AAV5-miHTT low | 8m | 44.41 | 5.04 | 24 | 0.8023 | 8m | 48.2125 | 8.025519758 | 24 | 0.9973 |
| AAV5-miHTT medium | 8m | 45.39 | 5.17 | 23 | 0.4798 | 8m | 50.36086957 | 4.509156254 | 23 | 0.6057 |
| AAV5-miHTT high | 8m | 44.85 | 6.53 | 25 | 0.6516 | 8m | 45.952 | 7.569958168 | 25 | 0.7933 |
| **Rotarod** | **Hu21** | | | | | **Hu128/21** | | | | |
| **Treatment** | **Age** | **Mean** | **SD** | **N** | **p value** | **Age** | **Mean** | **SD** | **N** | **p value** |
| Saline | 2m | 177.2307662 | 70.38326172 | 26 |  | 2m | 169.677421 | 71.05613517 | 31 |  |
| AAV5-miHTT low | 2m | 183.159998 | 74.16936119 | 25 | 0.9904 | 2m | 155.8484839 | 64.68106093 | 33 | 0.8683 |
| AAV5-miHTT medium | 2m | 172.7361183 | 63.94200421 | 24 | 0.9959 | 2m | 173.9166688 | 79.88984086 | 32 | 0.9955 |
| AAV5-miHTT high | 2m | 167.2133468 | 71.58722688 | 25 | 0.9566 | 2m | 164.1041675 | 71.38162196 | 32 | 0.9899 |
| **Treatment** | **Age** | **Mean** | **SD** | **N** | **p value** | **Age** | **Mean** | **SD** | **N** | **p value** |
| Saline | 4m | 96.10256231 | 63.38143746 | 26 |  | 4m | 61.35483987 | 53.25855591 | 31 |  |
| AAV5-miHTT low | 4m | 80.83999736 | 64.61245598 | 25 | 0.8246 | 4m | 38.43434224 | 50.36095003 | 33 | 0.3155 |
| AAV5-miHTT medium | 4m | 78.7638885 | 71.99553912 | 24 | 0.7676 | 4m | 52.39582953 | 62.10370114 | 32 | 0.9086 |
| AAV5-miHTT high | 4m | 68.8133336 | 51.69130701 | 25 | 0.4176 | 4m | 41.43749647 | 45.57724989 | 32 | 0.4483 |
| **Treatment** | **Age** | **Mean** | **SD** | **N** | **p value** | **Age** | **Mean** | **SD** | **N** | **p value** |
| Saline | 6m | 58.19999772 | 59.13731273 | 25 |  | 6m | 25.866668 | 29.72629078 | 30 |  |
| AAV5-miHTT low | 6m | 46.13333548 | 66.49457156 | 25 | 0.8345 | 6m | 23.156249 | 45.49724781 | 32 | 0.9878 |
| AAV5-miHTT medium | 6m | 40.59722321 | 43.18816051 | 24 | 0.619 | 6m | 15.73563117 | 29.5726626 | 29 | 0.6315 |
| AAV5-miHTT high | 6m | 21.81333304 | 21.66176894 | 25 | 0.0606 | 6m | 15.5416655 | 19.62474537 | 32 | 0.598 |
| **Treatment** | **Age** | **Mean** | **SD** | **N** | **p value** | **Age** | **Mean** | **SD** | **N** | **p value** |
| Saline | 8m | 56.09333548 | 64.96570129 | 25 |  | 8m | 18.68253819 | 29.8757508 | 21 |  |
| AAV5-miHTT low | 8m | 44.347225 | 77.03506632 | 24 | 0.8936 | 8m | 15.24999975 | 29.27452034 | 24 | 0.9661 |
| AAV5-miHTT medium | 8m | 30.68116191 | 53.88629721 | 23 | 0.4323 | 8m | 13.50724787 | 23.78712351 | 23 | 0.8981 |
| AAV5-miHTT high | 8m | 15.25333296 | 21.87660639 | 25 | 0.0684 | 8m | 11.19999996 | 12.7769327 | 25 | 0.7342 |
| **Open Field** | **Hu21** | | | | | **Hu128/21** | | | | |
| **Treatment** | **Age** | **Mean** | **SD** | **N** | **p value** | **Age** | **Mean** | **SD** | **N** | **p value** |
| Saline | 3m | 18.45306885 | 11.89570454 | 26 |  | 3m | 16.95243561 | 12.88879417 | 31 |  |
| AAV5-miHTT low | 3m | 16.43449033 | 12.40249425 | 24 | 0.9277 | 3m | 19.77393978 | 13.49235305 | 32 | 0.8004 |
| AAV5-miHTT medium | 3m | 16.58463971 | 9.68538576 | 24 | 0.9415 | 3m | 14.28407516 | 10.84449793 | 32 | 0.8258 |
| AAV5-miHTT high | 3m | 18.93093088 | 12.27464554 | 25 | 0.9989 | 3m | 17.73961463 | 11.93049757 | 32 | 0.9943 |
| **Treatment** | **Age** | **Mean** | **SD** | **N** | **p value** | **Age** | **Mean** | **SD** | **N** | **p value** |
| Saline | 9m | 8.12946208 | 8.629387126 | 25 |  | 9m | 10.22522405 | 5.914045185 | 20 |  |
| AAV5-miHTT low | 9m | 7.59926625 | 8.388151565 | 24 | 0.9989 | 9m | 4.892194095 | 5.112955601 | 21 | 0.591 |
| AAV5-miHTT medium | 9m | 14.40716057 | 16.78718549 | 23 | 0.3266 | 9m | 10.02113206 | 15.56871635 | 18 | >0.9999 |
| AAV5-miHTT high | 9m | 16.34575624 | 16.14768615 | 17 | 0.1771 | 9m | 16.20751191 | 20.23525194 | 23 | 0.476 |
| **NOL** | **Hu21** | | | | | | | |  |  |
| **Treatment** | **Age** | **T1 Mean** | **SD** | **N** | **T2 Mean** | **SD** | **N** | **p value** |  |  |
| Saline | 9m | 53.92772727 | 14.83707933 | 22 | 65.984 | 20.54205748 | 15 | **0.0227** |  |  |
| AAV5-miHTT low | 9m | 48.66761905 | 19.54718852 | 21 | 64.91777778 | 15.08058025 | 18 | **0.0034** |  |  |
| AAV5-miHTT medium | 9m | 50.02913043 | 16.67115034 | 23 | 63.46933333 | 18.32260761 | 15 | **0.0126** |  |  |
| AAV5-miHTT high | 9m | 53.14761905 | 19.88631311 | 21 | 68.32571429 | 19.25611892 | 21 | **0.0081** |  |  |
| **NOL** | **Hu128/21** | | | | | | | |  |  |
| **Treatment** | **Age** | **T1 Mean** | **SD** | **N** | **T2 Mean** | **SD** | **N** | **p value** |  |  |
| Saline | 9m | 53.0335 | 17.7112656 | 20 | 58.06 | 16.818471 | 13 | 0.2114 |  |  |
| AAV5-miHTT low | 9m | 52.33095238 | 15.53859804 | 21 | 57.62538462 | 23.20553656 | 13 | 0.2152 |  |  |
| AAV5-miHTT medium | 9m | 50.64764706 | 20.68555672 | 17 | 62.64363636 | 21.02196341 | 11 | 0.0742 |  |  |
| AAV5-miHTT high | 9m | 54.52857143 | 15.95484136 | 21 | 67.93133333 | 20.98215189 | 15 | **0.0182** |  |  |
| **Striatal volume** | **Hu21** | | | | | **Hu128/21** | | | | |
| **Treatment** | **Age** | **Mean** | **SD** | **N** | **p value** | **Age** | **Mean** | **SD** | **N** | **p value** |
| Saline | 9m | 13.40655769 | 0.531731725 | 13 |  | 9m | 12.69016 | 1.131985557 | 12 |  |
| AAV5-miHTT low | 9m | 13.42505467 | 1.22582189 | 15 | >0.9999 | 9m | 12.94894692 | 0.809267816 | 13 | 0.909 |
| AAV5-miHTT medium | 9m | 13.25041231 | 0.91294635 | 13 | 0.9779 | 9m | 13.10698143 | 1.050137389 | 14 | 0.6952 |
| AAV5-miHTT high | 9m | 13.06251643 | 1.100685638 | 14 | 0.8046 | 9m | 13.22721786 | 0.86503985 | 14 | 0.5001 |
| **Cortical volume** | **Hu21** | | | | | **Hu128/21** | | | | |
| **Treatment** | **Age** | **Mean** | **SD** | **N** | **p value** | **Age** | **Mean** | **SD** | **N** | **p value** |
| Saline | 9m | 32.04040308 | 1.619362199 | 13 |  | 9m | 32.48071462 | 2.911772985 | 13 |  |
| AAV5-miHTT low | 9m | 32.96176733 | 3.13452036 | 15 | 0.7605 | 9m | 32.01449077 | 2.426043411 | 13 | 0.9683 |
| AAV5-miHTT medium | 9m | 33.03485538 | 2.299712325 | 13 | 0.7365 | 9m | 32.15072571 | 2.782919167 | 14 | 0.9877 |
| AAV5-miHTT high | 9m | 32.08488929 | 2.487280699 | 14 | >0.9999 | 9m | 32.21205786 | 2.286644552 | 14 | 0.9932 |
| **CC volume** | **Hu21** | | | | | **Hu128/21** | | | | |
| **Treatment** | **Age** | **Mean** | **SD** | **N** | **p value** | **Age** | **Mean** | **SD** | **N** | **p value** |
| Saline | 9m | 3.506553846 | 0.283948736 | 13 |  | 9m | 3.290761846 | 0.278884106 | 13 |  |
| AAV5-miHTT low | 9m | 3.504178 | 0.355514203 | 15 | >0.9999 | 9m | 3.309882154 | 0.160080656 | 13 | 0.9974 |
| AAV5-miHTT medium | 9m | 3.380394769 | 0.276645022 | 13 | 0.7242 | 9m | 3.2792965 | 0.31870551 | 14 | 0.9994 |
| AAV5-miHTT high | 9m | 3.258865714 | 0.30121842 | 14 | 0.1706 | 9m | 3.156203929 | 0.22106845 | 14 | 0.5166 |
| **DARPP-32 IOD** | **Hu21** | | | | | **Hu128/21** | | | | |
| **Treatment** | **Age** | **Mean** | **SD** | **N** | **p value** | **Age** | **Mean** | **SD** | **N** | **p value** |
| Saline | 4m | 1 | 0.047770702 | 3 |  | 4m | 0.8903 | 0.033965129 | 3 |  |
| AAV5-miHTT low | 4m | 1.079966667 | 0.012546049 | 3 | **0.0306** | 4m | 1.077633333 | 0.023639021 | 3 | **0.0001** |
| AAV5-miHTT medium | 4m | 1.056633333 | 0.019857828 | 3 | 0.1312 | 4m | 1.0571 | 0.015420441 | 3 | **0.0002** |
| AAV5-miHTT high | 4m | 1.062166667 | 0.01423388 | 3 | 0.0929 | 4m | 1.0798 | 0.027627342 | 3 | **<0.0001** |
| **Treatment** | **Age** | **Mean** | **SD** | **N** | **p value** | **Age** | **Mean** | **SD** | **N** | **p value** |
| Saline | 9m | 1.000000769 | 0.189320119 | 13 |  | 9m | 0.949026354 | 0.210302639 | 13 |  |
| AAV5-miHTT low | 9m | 0.993111333 | 0.296875712 | 15 | 0.9998 | 9m | 0.9990683 | 0.191506772 | 13 | 0.942 |
| AAV5-miHTT medium | 9m | 0.936591538 | 0.258037817 | 13 | 0.9046 | 9m | 1.006674793 | 0.248640972 | 14 | 0.9106 |
| AAV5-miHTT high | 9m | 0.884005714 | 0.181670534 | 14 | 0.5893 | 9m | 0.923308843 | 0.244504282 | 14 | 0.9909 |
| **Lateral ventricle size** | **Hu21** | | | | | **Hu128/21** | | | | |
| **Treatment** | **Age** | **Mean** | **SD** | **N** | **p value** | **Age** | **Mean** | **SD** | **N** | **p value** |
| Saline | 4m | 1 | 0.276895708 | 3 |  | 4m | 1 | 0.562939832 | 3 |  |
| AAV5-miHTT low | 4m | 1 | 0.27542092 | 2 | >0.9999 | 4m | 1 | 0.04385052 | 2 | >0.9999 |
| AAV5-miHTT medium | 4m | 0.804535667 | 0.260835853 | 3 | >0.9999 | 4m | 1.053496667 | 0.560590412 | 3 | >0.9999 |
| AAV5-miHTT high | 4m | 1.204538 | 0.209796502 | 3 | >0.9999 | 4m | 1.860215 | 0.931350223 | 3 | 0.9966 |
| AAV5-GFP medium | 4m |  |  |  |  | 4m | 2.957131 | 1.142763754 | 2 | 0.8227 |
| AAV5-GFP high | 4m | 8.560244 | 1.764777305 | 2 | **<0.0001** | 4m | 8.060244 | 1.057670524 | 2 | **0.0017** |
| AAV5-miScr low | 4m | 1.5875985 | 0.571188837 | 2 | 0.9837 | 4m | 2.0967095 | 1.964367387 | 2 | 0.992 |
| AAV5-miScr medium | 4m | 2.071705333 | 1.257738138 | 3 | 0.6421 | 4m | 2.209967333 | 2.45749568 | 3 | 0.9713 |
| AAV5-miScr high | 4m | 4.032950667 | 0.069908006 | 3 | **0.004** | 4m | 1.790448333 | 1.728292918 | 3 | 0.9981 |
